# Effort inference and prediction by acoustic and movement descriptors in interactions with imaginary objects during Dhrupad vocal improvisation

**DOI:** 10.1017/wtc.2022.8

**Published:** 2022-07-05

**Authors:** Stella Paschalidou

**Affiliations:** Hellenic Mediterranean University, School of Music and Optoacoustic Technologies, Department of Music Technology and Acoustics, Greece

**Keywords:** Performance augmentation, Performance characterisation, Sensors, Real-time models, Control

## Abstract

In electronic musical instruments (EMIs), the concept of “sound sculpting” was proposed by Mulder, in which imaginary objects are manually sculpted to produce sounds, although promising has had some limitations: driven by pure intuition, only the objects’ geometrical properties were mapped to sound, while effort—which is often regarded as a key factor of expressivity in music performance—was neglected. The aim of this paper is to enhance such digital interactions by accounting for the perceptual measure of effort that is conveyed through well-established gesture-sound links in the ecologically valid conditions of non-digital music performances. Thus, it reports on the systematic exploration of effort in Dhrupad vocal improvisation, in which singers are often observed to engage with melodic ideas by manipulating intangible, imaginary objects with their hands. The focus is devising formalized descriptions to infer the amount of effort that such interactions are perceived to require and classify gestures as interactions with elastic versus rigid objects, based on original multimodal data collected in India for the specific study. Results suggest that a good part of variance for both effort levels and gesture classes can be explained through a small set of statistically significant acoustic and movement features extracted from the raw data and lead to rejecting the null hypothesis that effort is unrelated to the musical context. This may have implications on how EMIs could benefit from effort as an intermediate mapping layer and naturally opens discussions on whether physiological data may offer a more intuitive measure of effort in wearable technologies.

## Introduction

Unlike the causal relationship found in non-digital musical instruments, gesture-sound links in electronic musical instruments (EMIs) need to be artificially designed due to the separation of the control mechanism from the sound-producing engine. This may result in a shortfall in their expressivity, which has been attributed by d’Escriván (2006) to the rupture of the so-called “*efforted*-input paradigm”: without being dictated by the physical laws of mechanical couplings and with no requirement for energy exchange between actions and sound, designed mappings (Garnett & Goudeseune, 1999) leave space for interactions that may lead to an “unnatural” feeling of mis-match, such as the one that may occur when, for example, explosive sounds are controlled by minimal physical effort. It is especially an inherent problem of empty-handed interactions, which do not require physical contact with a control interface and thus lack a direct energy transfer link and any form of feedback that can be sensed through haptic receptors embedded in skin, muscles, and joints (O’Modhrain & Gillespie, 2018). This very problem has been also discussed by Waisvisz (in Wanderley & Battier, 2000), Vertegaal (1996), Winkler (1995), Ryan (1991), and Krefeld & Waisvisz (1990).

“Sound sculpting” in virtual musical instruments by Mulder (1998) is such an example, in which synthetic sounds are controlled by deforming (virtual) objects, as in stretching a rubber sheet, claying, carving, and chiselling. Despite the originality of Mulder’s intention in forming some kind of continuity with respect to the real world by taking advantage of our prior experience in using the body as a mediator between mind and matter (Leman, 2007),^1^ this group of instruments has had some critical drawbacks: the mapping was developed based on pure intuition and it relied only on the geometrical properties of the imagined objects, without accounting for the dynamical aspects of resistance the user would experience and the effort one would need to exert while deforming them had they been real.

Effort is a term used to describe physical or mental activity needed to achieve something (Cambridge University Press, n.d.) and is often regarded as an important aspect in music, “the impetus of musical expression” according to Bennett et al. (2007). However, systematic approaches to its role in music remain surprisingly limited. To this end, the current work proposes to inform mapping strategies of virtual musical instruments by drawing findings from well-established gesture-sound links pertaining to effort in non-digital music practices. The stance taken here is that by recording real music performances in ecologically valid settings, more robust gesture-sound links can be studied, ones that musicians have established as active practitioners over years of training rather than spontaneous responses to stimuli experienced by listeners as is often the case in designed experiments.

Hindustani (North Indian classical) singing—and the Dhrupad sub-genre in particular—offers a suitable case of music making for studying such links. While improvising, Dhrupad vocalists often appear to engage with their own singing by employing and manipulating imaginary objects with their hands in the air. They stretch, pull, push, throw, and execute other powerful movements with their hands in the air, all of which comprise gripping, pulling, and releasing phases (Rahaim, 2009). Although no real object is actually involved on the occasions of such gestures, singers move as if they are fighting against or yielding to some imaginary resistive force. The above observation suggests that distinctive patterns of change in acoustic features of the voice allude to the effortful interactions that such objects can afford through their physical properties, such as viscosity, elasticity, weight, and friction. This is additionally supported by vocalists’ frequent recourse to motor-based metaphors and mental images of forces. However, the link between hand gestures and the voice on the occasions of such interactions has not been studied before in a systematic way and is the subject of the current paper.

This work focuses exclusively on these repeated patterns of bi-manual effortful interactions, abbreviated to “MIIOs” (standing for “manual interactions with imaginary objects”) throughout this text. By drawing on theories of embodied (music) cognition (Leman et al., 2018; O’Regan & Noë, 2001; Varela et al., 1991) and by extending Gibson’s (1977) ecological theory of affordances to cover objects in the imagistic domain too, the current work examines the main assumption that musical thinking in Dhrupad singing is grounded in the ubiquitous patterns of actions we possess by interacting with the real world, and that when Dhrupad singers appear to interact with an imaginary object, the link to the sound resides in the interaction possibilities the object affords and the effort it is perceived to require.

To meet this end, the current work attempts to understand more about the functionality of MIIOs by examining how much effort each of them is perceived to require, and whether and how MIIOs and the exerted effort are related to their melodic counterpart or if they just co-appear arbitrarily. In fact, for this task it is essential to examine to what extent such gesture-sound links are consistent across performers or rather are idiosyncratic. It is also required to disambiguate between effort that reflects conceptual aspects of melodic organization from the straightforward mechanical requirements of vocal production.

## Background

This section aims to clarify the way effort is understood in this study, to justify the selection of Dhrupad singing as a case study for proposing enhancements in EMI mappings as well as the choice of acoustic features for the quantitative part of the analysis, and finally to outline the original contribution offered by this work.

### Effort

Effort in its reserved, everyday use reflects our understanding of how hard a person must try in order to achieve an intended goal. In other words, it refers to the forcefulness or power of an action and it stresses the importance of intentionality and compulsion. Still, it seems to be a loosely defined term in different branches of science, such as physiology, kinesiology, biology, neuroscience, and psychology (Massin, 2017; Richter & Right, 2014). The reason for this lies in its perceptual and subjective character (Steele, 2020) and in the compound nature of the goal (Dewey, 1897), consisting of both overt (physical) and covert (cognitive) aspects, which make effort difficult to grasp and quantify.^2^

In psychology and neuroscience, mental (or cognitive) effort is a concept that represents the subjective sense of cognitive strain (Westbrook & Braver, 2015) required for a given task and has been associated with mental imagery (Papadelis et al., 2007) and the level of attention (Bruya & Tang, 2018; Mulder, 1986; Kahneman, 1973). Mental effort is shaped in the form of peaks and decays or impulses and rebounds, which are also supposed to have emerged from our general capacity to move through approach and withdrawal (Stern, 2010).

In the realms of dance, choreography, and kinesiology, effort is considered a subjective measure of expression used to describe qualities of movement performed with respect to inner intention. It is associated with the forces that cause and constrain movement—the active or passive attitude of a person in resisting or yielding to the physical conditions that influence a movement, that is, its dynamics—and at the same time it reflects the inner impulse or source of the movement—the inner attitude of power in terms of motivation and intentionality—that is harnessed by a person to accomplish an intended goal (Maletic, 1987; Bartenieff & Lewis, 1980; Laban & Ullmann, 1971). It “exposes the mover’s manner, tone and level of energy” (Hackney, 2002) and features recognizable “motion bell” patterns (Camurri et al., 2003) of two consecutive distinct phases—tension/exertion and release/relaxation—that shape and phrase expressive physical action. Most notable in movement studies has been Laban’s movement analysis, a system that Laban developed for categorizing movement in the context of Western modern dance choreography (Laban & Lawrence, 1974).

In the acoustic world, musical effort has been described as “the element of energy and desire, of attraction and repulsion in the movement of music” (Ryan, 1991: p. 6). It has been reported that it reflects the musical tension of a piece (Cox, 2016; Krefeld & Waisvisz, 1990), which is manifested as patterns of musical intensification and abatement (Kurth, 1922) giving rise to emotional responses (Lerdahl & Krumhansl, 2007). The notion of effort has been appreciated as a basic feature of expressive power in EMIs (Wanderley & Battier, 2000; Ryan, 1991) and is considered as essential to both performers and audiences alike (Olsen and Dean, 2016). Performers need to suffer and even sweat a bit in order to bring this tension to the fore and audiences need to perceive effort in order to recognize particularly intense musical passages played by the musician (Vertegaal et al. 1996); in fact, much of the excitement in audiences is often driven by levels of virtuosity and effort observed in performers. Thus, effort in music can be conveyed and induced by a performer, as well as assessed and experienced by an observer. It can be visible in human movement, audible in the sound, and sensed through proprioception.

While in movement action is directed toward a designated pragmatic goal (Rosenbaum et al., 2009), in music the goal is primarily musical and refers to salient moments of musical expression that are regulated or accompanied by music-related gestures. Hence, effort in music can combine bodily and melodic movement (overt), mental impulse, and musical intentionality (covert). Nevertheless, none of the descriptions or definitions has captured the term in its entirety and none has acknowledged sound as part of both its overt (auditory) and covert (imagery) aspects. Therefore, a working definition of effort in music—one designed specifically for the purpose of this study—might combine and extend previous definitions to not only include movement but also the auditory modality:

Effort constitutes a perceptual and subjective measure that combines the covert (musical imagery) and overt (movement & sound) aspects of power and intentionality put towards a goal in reflecting the active or passive attitude of a person in fighting against or giving in to the compound forces or conditions that might resist the accomplishment of the task.

In MIIOs, effort can be imagined and experienced by the performer and perceived (visually and auditorily) by an observer. Dhrupad vocalists seem to manipulate notes by grasping, holding, extending, restricting, and releasing an object, which signifies successive intensification and relaxation phases. Thus, effort is expressed through the subjective quality of balance between, on the one hand, the imagined resistive force imposed by the object that is employed by the performer and, on the other hand, the force that the performer exerts to defy it. It is a measure for the power of this interaction and can be potentially regarded as a cross-modal (or amodal) descriptor that combines all implicated modalities (sound, vision, movement, mental imagery). This highlights the originality of the multimodal feature fusion method in the regression analysis—described in detail in the Methodology—to efficiently combine movement and acoustic features into a common effort representation. It also justifies the sequential mixed methodology followed, which combines interviews, audio-visual material, and movement data.

The assessment of effort relies commonly on the subjective experience of how difficult the compound task seems to be for the person actively involved or observing, and therefore effort (in music too) is a perceptual measure that does not offer straightforward ways to quantify it. This might explain why analytic works showing a clear focus on the concept of effort with this specific understanding are limited and why there is a shortage of successful quantitative methods and measures, especially those looking at music performance.

For instance, tension has been studied extensively in music (melodic: Granot & Eitan, 2011; Bigand & Parncutt, 1999, vocal: McKenna & Stepp, 2018), but it is a complex phenomenon to quantify and its validity as an indirect measure of effort is not well-justified in literature. Laban’s effort ideas have proven valuable for developing computational methods and have laid the foundations for the majority of work on the analysis (Glowinski et al., 2017; Fdili Alaoui et al., 2017; Broughton & Davidson, 2014; Maes et al., 2014; Camurri et al., 2009; Petersen, 2008) and synthesis (Volpe, 2003; Zhao, 2001) of movement in music, as well as the development of interactive systems for music and dance (Souza & Freire, 2017; Françoise et al., 2014; Maes et al., 2010; Bennett et al., 2007). However, his ideas have limited potential for developing explicit quantitative measures, and in the abovementioned computational methods Laban’s effort qualities are only indirectly estimated by extracting features that are usually evaluated as relevant to the concept of effort by observers.

It can be tempting to approach effort through force-related measures borrowed from physics and mechanics, such as “weight” (not necessarily in the vertical direction), “power,” or “pressure” (Moore & Yamamoto, 2012), as they reflect the action’s power or the ease and struggle in performing an action. “Weight” has been indirectly computed by extracting measures for acceleration (Niewiadomski et al., 2013; Kapadia et al., 2013; Samadani et al., 2013; Castellano & Mancini, 2009; Caridakis et al., 2007; Bernhardt & Robinson, (s. d.); Hartmann et al., 2005; Volpe, 2003; Camurri et al., 2003) and kinetic energy (Piana et al., 2013; Samadani et al., 2013; Hachimura et al., 2005; Nakata, 2002) or “Quantity of Motion” (Mazzarino et al., 2009) of the whole body or different body parts (other indirect measures have been also probed, for example, Mentis & Johansson, 2013; Zhao & Badler, 2005; Morita et al., 2013; Alaoui et al., 2012).

However, there is a lack of general agreement on how to compute “weight” (Niewiadomski et al., 2013), which has even led to avoiding it entirely (Santos, 2013; Camurri and Trocca, 2000). First, it is worth emphasizing that “weight” should not be assessed through measures that include kinetic properties (kinetic energy, acceleration, and velocity)—in other words measures that reflect the impact of one’s movement (Chi et al., 2000). These would fail, for instance, to represent effort exerted in trying to move an object fixed in space (such as a wall) or in holding a heavy load for a long period of time without moving. More importantly, studies approaching effort through force-related measures do not acknowledge its compound character (comprising both physical and mental aspects) and additionally presuppose that the sense of an action’s power can be somehow objectively assessed and quantified. Since this is not necessarily the case, this assumption needs to be further examined. Considering that different people have a different capacity (the intrinsic factor related to a person’s level of fitness or ability) in achieving the same physically and/or mentally demanding goal (the extrinsic factor related to the task’s difficulty level), effort constitutes a perceptual concept, related to the subjective sense of how intense the specified task is for the particular subject.

A few approaches exist that rely on the physiologically measurable quantities that are supposedly the prevailing indicators of effort in music, such as muscular tension, breathing rate, biochemical energy expended in form of heat (calories), sweating, pupillary dilation, and brain activity (Jagiello et al., 2019; O’Shea & Moran, 2018; Ward et al., 2016; Tanaka, 2015; Gibet, 2010; Marcora, 2009; Enoka & Stuart, 1992; Kahneman, 1967). However, these are absolute measures that do not necessarily account for the capacity of a person in executing the task and may not reflect the subjective sensation of effort, therefore they do not hold wide consensus. More systematic work needs to be done in this direction. For instance, the absolute measure of force may be low, but due to low muscle power or fatigue, burned calories may exceed the resting burn rate (measured through metabolic equivalents). Additionally, the compound character of effort (comprising both physical and mental aspects) would require capturing a combination of physiological measures from different modalities, which is usually not the case. The shortage of successful quantitative approaches underlines the original contribution of this study in combining qualitative with quantitative methods.

### Effort in EMIs

Archetypical interactions with objects of the real world that we are familiar with may offer an easily accessible interaction metaphor for both novice and expert music performers of novel empty-handed EMIs (Bevilacqua et al., 2017; Ward, 2013; Essl & O’Modhrain, 2006; Fels et al., 2002). However, most digital-music interfaces do not offer “ergotic” gesture-sound couplings (Castagné et al., 2004); although the automatic tendency for effort minimization has been evidenced in multiple fields (Cheval & Boisgontier, 2021) and while effortless movements may be perfectly suited for word processor design, they may have no comparable utility in the design of musical instruments (Ryan, 1991: p. 3). There is extensive evidence that effort can add value and therefore be opted against actions that are effortless (Inzlicht et al., 2018). Together effort and sound reinforce one another, and their strong link increases expressive power and the likelihood of the EMI to last over time (Fels et al., 2002). For instance, Fels et al. (ibid.) have reported that while claying was not considered a compelling metaphor for shape manipulation due to the absence of tactile feedback, the stretching metaphor proved to compensate for this absence by forcing the player’s frame of reference to remain attached to the object.

Therefore, there is a number of indicative approaches (Battey et al., 2015) that consider how haptic- (especially force-) feedback interfaces could be used as a means for improving the control accuracy of synthetic sound, for example, pitch-selection accuracy on a continuous pitch controller (Berdahl et al., 2009), which is especially crucial when many degrees of freedom need to be managed (Wisneski & Hammond, 1998). In empty-handed interactions, as in the case of MIIOs, it may be possible to compensate for the absence of tactile feedback by encouraging users to imitate the exertion of effort against forces that are elicited by physically inspired images of interactions with real objects and restricting patterns of sonic qualities to only the ones that the implicated materials can afford. By using gesture-sound mappings that conform to the amount of required effort elicited through images of interactions, such as those of MIIOs in Dhrupad singing, the intention is to render the control of artificial sounds more natural, intuitive, and “physically plausible” (Castagné & Cadoz, 2005) and contribute to enhancing EMIs in terms of controllability, expressivity, and virtuosity (O’Modhrain & Gillespie, 2018; Yu & Bowman, 2018; Tanaka, 2015; Ward, 2013; Essl & O’Modhrain, 2006).

Sensations of elasticity, weight, and other physical metaphors in musical thought are not exclusive to Hindustani music, and listeners and performers in other musical genres also report that they experience virtual worlds of forces and motion in relation to music and sound (Fatone et al., 2011; Eitan & Granot, 2006). From an engineering point of view, Mion et al. (2010) have in fact modeled gesture-sound links in the context of instrumental gestures based on the concept of resistance or impedance. However, Dhrupad vocal music constitutes a distinctive case in this sense for reasons which are explained next. A short introduction to Dhrupad and MIIOs is first given.

### Introduction to Dhrupad

Dhrupad (Widdess & Sanyal, 2004) is one of the two predominant styles of Hindustani music. It is a monophonic (primarily) vocal music tradition, and it relies heavily on improvisation, called ālāp, which is not free but strictly rule based and conforms to the so-called rāga system (a form of melodic mode lying between scale and tune; Powers & Widdess, 2001). The tonic is defined by the main performer’s most comfortable pitch and all other pitches are tuned relative to this. The improvisation starts at a strikingly low pace in the vocalist’s lowest pitch range; it has a very slow melodic development, which builds up only gradually in pace, pitch, and melodic tension as the melody ascends over about 2.5 octaves toward the climax (typically the 3^rd^ degree of the highest octave) and it is sung without apparent rhythm (non-metered) to a repertoire of non-lexical syllables (e.g., “ra,” “na,” and “num”). Melodic tension is periodically released by longer stops on the tonic and shorter stops on the 5^th^ and is finally resolved when the climax is reached.

### MIIOs in Dhrupad

Hindustani singers seem to engage with the melodic content in two main ways (Rahaim, 2009), which are supposed to reflect two different modes of body–voice relationship: the open handed and the closed handed. In the closed-handed mode, which starts with forming a grip with the hands as in grasping an object, singers look like they are stretching or compressing an elastic material, pulling or pushing away a heavy object, throwing or bouncing something like a ball on the floor, and executing other powerful movements in changing the objects’ shape, size, and position, all of which comprise gripping, intensification, and releasing phases.

**Figure 1.**
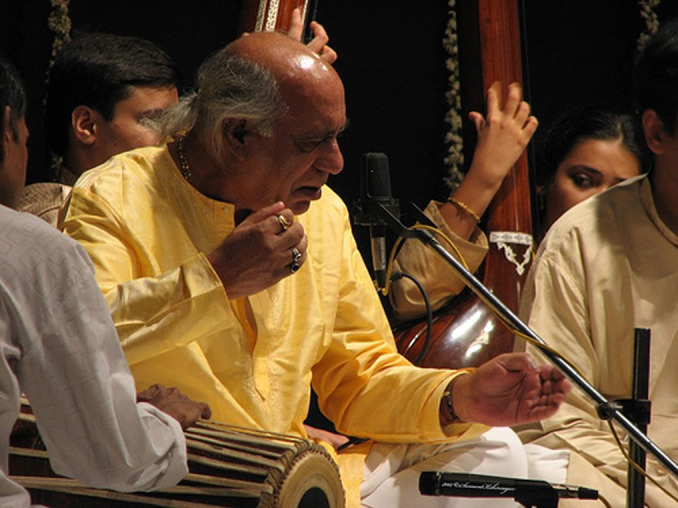
Zia Fariduddin Dagar in concert, 16.06.2007 (Mana, 2007).

In contrast to the open-handed mode (ibid.), in which the hands seem to trace curves and trajectories effortlessly in space, the powerful movements of the closed-handed mode do not seem to offer a simple melographic representation of the sound (spatial representation of pitch height) and do not carry any symbolic meaning, but rather they appear to indicate proprioceptive sensations of resistance that singers employ in manipulating notes as smooth pitch glides; Figure 1 illustrates such an example. There is an observable match between the voice and the manipulative gestures in terms of synchronization and temporal congruency of various features, mostly melody and melodic tension, dynamics, and timbre and—despite the absence of a real object—the link to the sound seems to be mediated by the imagined material.

From the point of view of enactive theories and ecological psychology, this match attests to the importance of sensorimotor skills or patterns of robust movement-sound contingencies (“know-how”) that a person develops over time by manipulating real objects of the environment (Freed, 1990; Warren & Verbrugge, 1984; Gibson, 1977). These patterns underpin much of our conception of any sound (Maes et al., 2014, Küssner, 2014; Cox, 2011; Zbikowski, 2002), thus we attend any sonic event—be it real or virtual (Clarke, 2005)—with certain expectations (Huron, 2006) through an incessant process in the imagistic domain of mentally simulating features of sound or actions that may have produced it (Cox, 2011; Lahav et al., 2007). As it has been suggested that we are able to grasp objects figuratively because we are able to grasp them literally (Küssner, 2014), it would not be a crude assumption to also think of an imagined object in MIIOs as a carrier of certain universal patterns, general rules, and opportunities for behaving (Camurri et al., 2001) that are defined by the imagined resistive force according to size, shape, material, and so forth and that also afford specific types of sonic results (Krueger, 2014; Tanaka et al., 2012; Menin & Schiavio, 2012; Reybrouck, 2012; Godøy, 2009; Nussbaum, 2007; Jensenius, 2007; Clarke, 2005). However, the link between hand gestures and voice in MIIOs has not been studied before in a systematic way in respect to the concept of effort.

### Dhrupad as Case Study

Singing offers a unique opportunity to study profound gesture-sound links that are driven by the musicians’ mental constructions and musical intentions, as—in contrast to instrumental playing—the hands are not bound by the mechanical constraints posed by a particular instrument. In fact, it is quite surprising that singing gestures have drawn less attention than instrumental ones (Pearson 2016; Clayton & Leante, 2013; Fatone et al. 2011; Rahaim, 2009; Moran, 2007). Even more, the spontaneous and easily identifiable gestural demonstration of physically inspired concepts by MIIOs is not common in the performance practice of music genres other than Dhrupad, making it unique in allowing an ecologically valid methodological approach in recording performances without any explicit instructions like those required in designed experiments. It also imposes constraints upon musicians’ movements that reduce the otherwise high complexity of full-body motion and the necessity for the use of artificially induced exogenous constraints or dimensionality reduction methods (Nymoen et al., 2013).

The conception of melody as a continuous pitch “space”^3^ (Fatone et al., 2011), in which Dhrupad vocalists often approach discrete notes through smooth trajectories rather than simply scale steps (Battey, 2004), makes Dhrupad particularly suitable for studying links to the non-discrete nature of movement and for enhancing mapping strategies of EMIs in which performers’ gestures serve the continuous control—monitoring and adjustment—of patterns generated by computer algorithms rather than the triggering of individual events. The precise intonation of Dhrupad musicians and the slow rendering of melodic phrases in the opening section of the ālāp improvisation make it ideal for studying MIIOs, as for slow musical stimuli there is a tendency to ascribe motion to the impact of an outside force on an imagined character (Eitan & Granot, 2006). The lack of meter and meaningful lyrics is suited to focusing on melodic factors and the expressive rather than semantic content imprinted on gestures. Additionally, Dhrupad offers a rigid framework for the study of melodic tension, in terms of both specified ālāp improvisation-relevant macro-structure (gradual intensification from lower pitches toward the climax) and rāga mode-specific micro-structure (locally raised tension for specific melodic movements according to the rāga “topography” (Rahaim, 2009)).

As Dhrupad is an oral music tradition, knowledge is not transmitted through music notation but through direct demonstration and imitation, which includes not just sound but also movement. The resulting gestural resemblance that is evident between teacher and students (Rahaim, 2009) justifies the deliberate choice of collecting material from a single music lineage and permits examining similarities (inherited bodily disposition) versus differences (idiosyncrasy) in the gesturing habits of musicians who share the same teacher. Finally, interviewees’ frequent recourse to motor-based metaphors allows a mixed—thus richer—methodological approach to be followed, one combining measurements with interviews (Leante, 2009).

Computational approaches of cross-modal relationships for singing movements have been few in Western types of music (Brunkan, 2015; Pfordresher et al., 2015; Luck & Toiviainen, 2008) and even fewer in Indian music (Pearson, 2016; Moran, 2007). Even more, none of the work has focused solely on MIIOs, and specifically in Dhrupad, none has applied quantitative methods through such an unusually strong ecological validity in the capture of data in the field, including 3D-movement, and, most importantly, none has acknowledged the role of effort as central to the subject of music performance and approached it in a systematic way. Although regression has been previously used in gesture-sound studies (e.g., Caramiaux et al., 2009), to the best of our knowledge this is the first time that the compound—and therefore complex—character of effort has been considered by combining both movement and acoustics features in each single model for inferring and predicting effort (with preliminary results first presented in Paschalidou et al., 2016). Despite the specificity of the genre that is being used here as a case study, this study aims to address concerns that are of interest to the wider research community and extend outcomes to other music-making practices and new EMIs in specific.

## Data

The material of the current paper is based on original recordings that were made for this study during fieldwork in four different cities in India in 2010–2011, comprising interviews, audio-visual material, and 3D-movement data from Dhrupad vocal improvisations. Seventeen professional and amateur vocalists of both genders, different ages, levels of musical experience, and years of training were recruited, only two of whom were not Indian. For consistency reasons in terms of gestural resemblance, all selected participants belong to the same music lineage; in specific, they are disciples of the renowned vocalist Zia Fariduddin Dagar, who himself was also recorded. Of the 17 musicians who were recorded singing, 8 were interviewed, all senior performers. Participants were informed beforehand only about the fact that the study was related to music and movement, but no further details were given. All musicians were paid a small amount per hour for the recording of performances but not for interviews.

**Figure 2.**
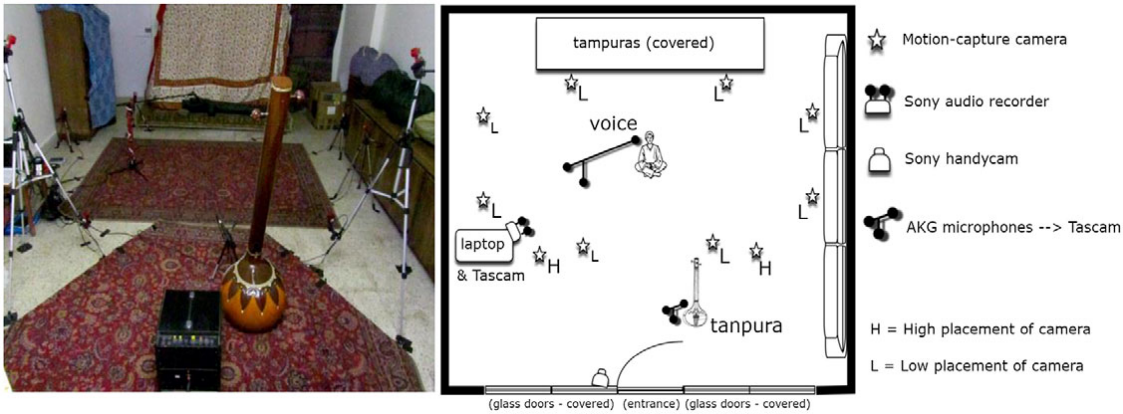
Typical equipment setup, photo and layout from Palaspe/Panvel, school of Zia Fariduddin Dagar.

To ensure ecological validity, musicians were simply asked to perform an improvisation in a rāga of their own choice without further instructions. Recordings varied in length according to the willingness and mood of the musicians to sing, as well as the peculiar recording conditions (unexpected power cuts, ambient noise, other scheduled activities), ranging between 10 and 70 min. Some musicians were recorded only once, while for others more than one session was possible, giving a total of about 18 h of recordings. All recordings were conducted in domestic spaces, usually the living room in which musicians hold their daily musical activities, these rooms adapted to meet the needs of the recordings as illustrated in Figure 2. Recording equipment included:

1. TASCAM HD-P2 audio recorder and a paired AKG C 480 B set of two microphones for the voice and the drone instrument that supports vocal tuning (*taňpurā*), set at 192 kHz, 24bit;
2. Sony PCM-D50 audio recorder for ambience, set at 96 kHz, 24bit;
3. Naturalpoint Optitrack passive marker-based optical motion capture system with 10 high-speed infrared cameras and a 3.5″ (wide) optical angle lens, set at 100 fps;
4. Sony DCR-SR65 handy-cam recording in night shot (infrared) from a fixed position.

To limit arbitrary reflections that caused confusion in tracking the markers, recordings were made in low lighting conditions and most furniture was removed from the space or covered. Video was recorded in night shot and visual contact was enabled by a dim light. The motion capture system was calibrated prior to each recording session. Hand claps were used for the purposes of syncing independent data streams; they were carried out by the performer or the author every 10 min by wearing reflective markers on the hands. Skeleton reconstruction was based on the centroids of 13 distinctive rigid bodies, that is, groups of markers forming unique geometrical shapes. With the exception of the head (4), rigid bodies consisted of 3 markers each and included the tracking of 2 fingers on each hand by custom-made markers protruding on rings.

## Analysis

### Methodology

**Figure 3.**
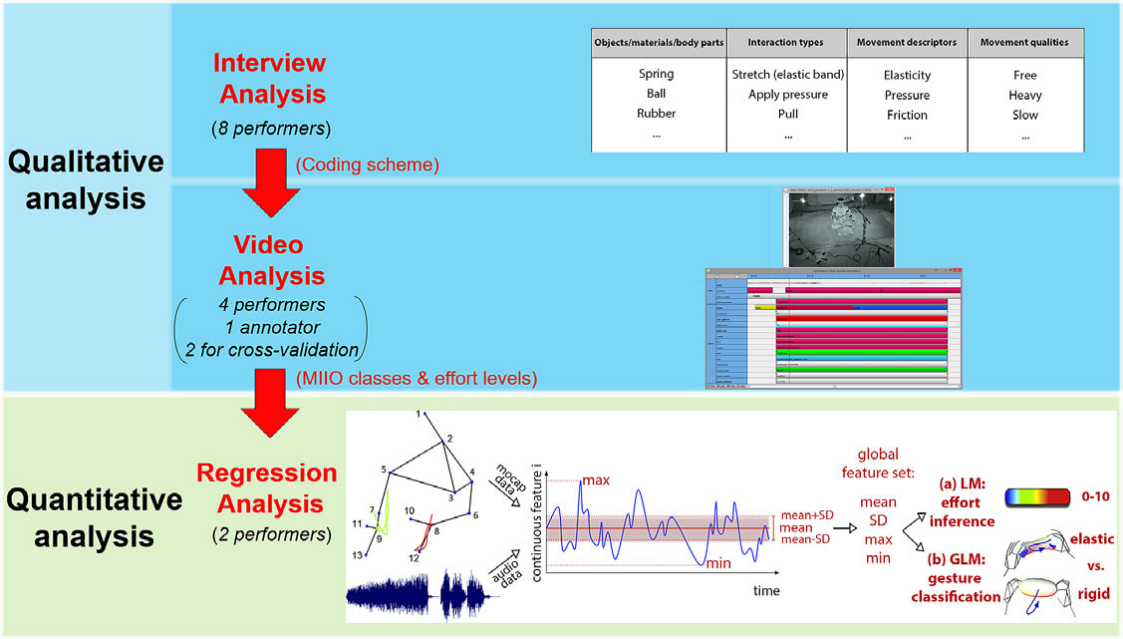
The sequential mixed methodology that was followed. Gesture images taken from Mulder (1998).

Due to the explorative character of the work, an empirical sequential mixed methodology was followed, which relies on first- and third-person perspectives for gesture analysis in music (Leman, 2010) and combines qualitative ethnographic methods (thematic analysis of interviews and video observation analysis of vocal improvisation performances) with quantitative methods (regression analysis of the same performances) on original recordings of interviews, audio-visual material, and 3D-movement data. The qualitative part of the work preceded, informed, and guided the quantitative methods that followed. The entire methodology is briefly outlined here and illustrated in Figure 3; however, the current paper reports exclusively on results drawn from the quantitative methods used. The reader can refer to Paschalidou & Clayton (2015) for a brief outline of the qualitative analysis, which clearly revealed a significant visual element in the conceptualization of music and a frequent use of sensorial descriptors and metaphors of interactions with objects.

For the interviews, thematic analysis was applied to motor-based metaphors and physically inspired linguistic descriptors of 8 performers, which were coded and organized in meaningful ways in classes of overarching themes into a final table intended to inform the coding scheme of the video analysis that followed. For the non-participant observation analysis, the audio-visual material of the vocal improvisation by four performers was manually annotated by a single observer, who visually identified, segmented, and labeled ΜΙΙΟ events and coded them for a number of melodic and movement aspects, most importantly:

- effort levels (numerical) that each gesture was perceived by the observer to require, in an integer scale of 0–10 (10 being the highest) and
- MIIO classes, such as stretching, compressing, pulling, pushing-away, collecting, and throwing (categorical descriptors).

These manual annotations were cross-validated by two choreographers by conducting an inter-coder agreement test and were then used as the “true” response values in fitting simple, interpretable linear models (LMs) with a reasonable accuracy to a small number of combined movement, and audio features extracted from the raw data of two vocal improvisation performances (which were selected from the entire dataset based on the high number of identified MIIO events). Specifically, regression analysis aimed to validate, augment, and formalize findings from the qualitative methods by avoiding manual labor and inferring annotations computationally with the following intention:

1. to test the null hypothesis that physical effort and MIIO types are unrelated to the melodic context against the alternative hypothesis that such a relation does exist and
2. to devise formalized descriptions by probing various sets of statistically significant movement and acoustic features for two different tasks:
3. to infer effort levels
4. to classify MIIOs.
  Different models were devised for each task, (i) those that best fit each individual performer (thus reflect mostly idiosyncratic elements of each individual performer) and (ii) those that—despite their lower goodness of fit—overlapped to a greater extent across performers in terms of number and type of features (thus describe more generic gesture-sound links).

There are several challenges in applying traditional statistics by fitting linear regression models to time-varying data as the ones examined in this paper: data not following a normal distribution, limited dataset not allowing the separation between training and testing, issues of data temporal dependency (Schubert, 2001), and non-linear elements that are not captured by the fitted LMs (Stergiou, 2011). However, linear regression offers the unique advantage of illustrating a rather complex phenomenon of the real world in a compact and easily interpretable way through a model that allows one to verbally describe a relationship in simple terms. The first part of this work seeks to gain a deeper understanding of the Dhrupad performance practice on the occasions of MIIOs based on the relatively limited size of the originally recorded dataset; therefore, regression analysis was considered as a suited first step and was opted for this study in proposing enhancements to EMI gesture-sound mappings.

### Response Values

For inferring effort, the full range of integer values assigned by the annotators was used for the models. However, for the classification task the five fine MIIO classes by which the material was originally annotated were reduced through simple observation of the video footage to only two coarse categories, specifically discriminating between interactions with (a) elastic versus (b) rigid objects. This reduction was a requirement for running multinomial (logistic) regression models (response classes > 2) with a small sample size (64 for vocalist Hussain and 102 for Sahu) and working with skewed samples of real performances rather than designed experiments. Interaction with elastic objects refers to the deformation of a material with elastic properties by extending or compressing it from its equilibrium (as in stretching/compressing), while interaction with rigid objects refers to the spatial translation of a non-malleable body of some considerable mass (as in pulling/pushing away/collecting/throwing). Visual discrimination by the annotator between these two classes was led by the dynamic profile of the gesture events and the existence or not of a subsequent recoil phase toward the equilibrium. Ambiguous classes were excluded.

Table 1 summarizes all relevant information about the data that were used for each of the two performances and illustrates the correspondence between fine and coarse gesture classes.

**Table 1.** Data overview for vocalists Afzal Hussain and Lakhan Lal Sahu

	ālāp improvisation	
Vocalists	Afzal Hussain	Lakhan Lal Sahu
Setup	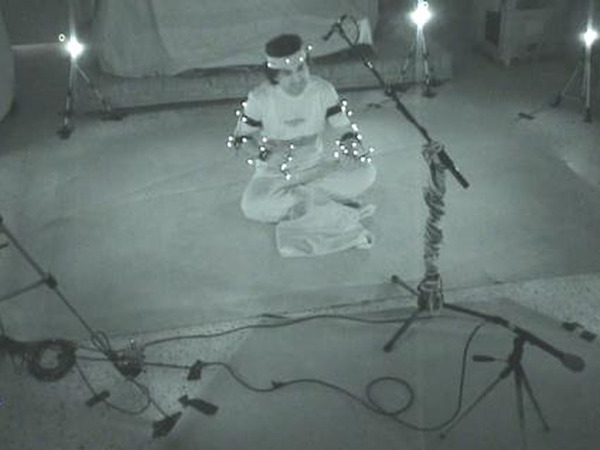	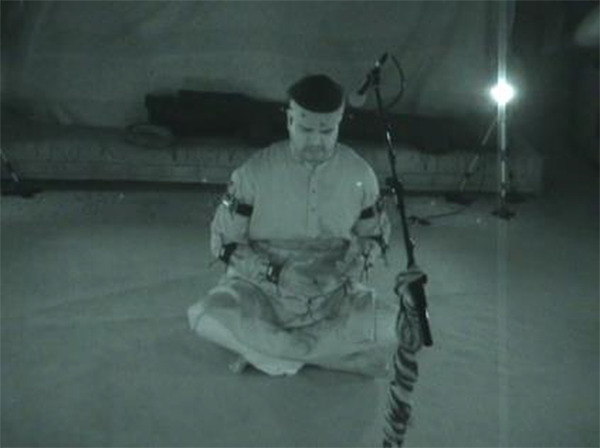
Performance details	rāga mode: Jaunpurī (Autrimncpa, 2012a) 45 min total duration 18 min ālāp improvisation	rāga mode: Mālkaunś (Autrimncpa, 2012b) 33 min total duration 23 min ālāp improvisation
Total MIIO events	64	102
Elastic objects	18 (28.1%) Stretching, pushing to compress	72 (70.6%) Stretching, pushing to compress
Rigid objects	46 (71.9%) Pushing away, pulling, throwing, collecting (or taking)	30 (29.4%) Pushing away, pulling, collecting (or taking)

### Feature extraction

Raw mocap data were exported into c3d format and were processed by:

1. Filling gaps that are caused by occasional optical occlusion of reflective markers, through linear interpolation between adjacent points in time and
2. Smoothing to eliminate noise introduced by arbitrary reflections in space and occasional confusion in identifying the rigid bodies, through a low-pass Savitzky–Golay FIR filter of 9^th^ order with a window of 90 msec for position and velocity data, and 130 msec for acceleration data.

Representative statistical *global* measures (such as mean, standard deviation (SD), minimum (min), maximum (max)) were computed from *time-varying* movement and audio features (Matlab 2015: Mocap Toolbox v. 1.5/MIR Toolbox v. 1.6.1, Praat v. 6.0^4^) and were normalized prior to their use in the models. Due to the endless possibilities in the quest for the best combination of features, this work started from a basic set of features that has been previously proposed in a different experimental context (Nymoen et al., 2013), as basic descriptors have proven robust in sound tracing experiments (ibid.) and violin bow strokes (Rasamimanana et al., 2006). This was then progressively enriched with alternative or novel features considered more relevant to the specific study and performance context and expected to raise the explained variance in the estimated responses.

For instance, to account for the different ways by which Dhrupad singers seem to display similar qualities of movement in a real performance setting without any instructions, hand distance was computed according to handedness (uni- or bi-manual), that is, the active hand(s) participating in the movement (rather than simply hand distance as in the basic feature set), and the point of reference to which gestures appear to be performed (according to gesture type this can be the position of the other hand, the torso, or the starting point of the movement and features are designated by the suffix “*_accord”*), which were both drawn from the manual video annotations. Additionally, it made more sense to compute position features in relation to the centroid of the torso (point 3 in Figure 3) rather than the ground, which was used in the basic feature set, because not only gravity but other forces are imagined acting too and Dhrupad singers often conceptualize pitch as a movement in relation to their own body.

**Figure 4.**
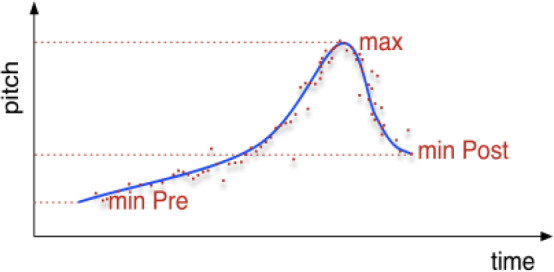
Critical pitches defined by three values (minPre-max-minPost) for double-sloped melodic glides.

Pitch-related features for the three most typical melodic movements in Dhrupad (ascending, descending, double-sloped) were computed in three alternatives: (a) the values of critical pitches (minPre-max-minPost) as shown in Figure 4, (b) the coefficients of polynomial expressions (of 2^nd^–5^th^ degree) that were fit to pitch data and forced to pass through the critical melodic points, and (c) the asymmetry in strength and rate of pitch ascent versus descent (calculated in a similar way to the sum of signed vertical kinetic energy of a unit mass in the basic set; Nymoen et al., 2013). Critical pitches were extracted in three different scales: linear, absolute logarithmic, and relative logarithmic (designated by the suffix “*_lin,”* “*_abslog,”* “*_rellog”*). Absolute logarithmic values refer to the absolute pitch height and thus they better reflect the mechanics of voice production, while relative logarithmic values refer to degrees of the scale in relation to the tonic of each octave and thus they are assumed to better reflect the melodic organization that is specific to the rāga mode:

- linear: 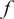 in Hz,
- absolute logarithmic: 
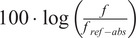
 ,
- relative logarithmic: 
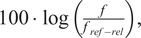

where *f_ref-abs_* = 440 Hz and *f_ref-rel_* = the tonic of the specific octave in which the melodic movement takes place (drawn from manual annotations).

### Determining Best Models

The best models were determined (R v. 3.2.3/R-studio v. 0.99.491) by trial-and-error in finding the best trade-off between accuracy of model fit, compactness (small number of independent variables), and simplicity in interpretation and feature extraction. Apart from the best-fitting models to a single performer, slightly less successful but largely overlapping cross-performer models were also devised, representing more generic cross-modal relationships. Whether these models can be potentially generalized to a wider population is currently unknown; however, they are useful in indicating trends that can be further explored in larger datasets. As methods were mostly intended for inference (and less for prediction) and the dataset that followed from the use of uninstructed real performances rather than designed experiments was limited, the entire dataset was used for fitting models (no separate training data were used for prediction).

The goodness of fit, according to which the best models were selected, was assessed based on the adjusted R-squared (*R^2^adj*) coefficient of determination (James et al., 2013: 212) for the LMs in inferring effort and on the area under the curve (*AUC*) of the receiver operating characteristic curve (ibid.: 147) for the general logistic models (GLM) in classifying MIIOs. The strength and the direction of the correlation of each individual feature to the response were rated based on the absolute value and the sign of each individual coefficient and the probability (*p*) value was used as an indication of likelihood in observing an association between feature and response due to pure chance in the absence of any real association. A significance level α of 0.05 (Moore & McCabe, 2006) was used in comparison to the *p*-value for examining the null hypothesis.^5^

## Results

The initial feature set produced poor results, while a replacement by alternative non-collinear features (two or more explanatory variables that are not strongly correlated with each other) increased the fit dramatically for both effort level estimation and gesture classification. All feature name abbreviations that appear in the models are explained in the Appendix (Table A1), while the reader can refer to Paschalidou (2017) for a full list of all features that were probed as potential candidates in the quest for successful models. Audio feature names appear in italic, while movement features are in plain text.

### Effort Level Estimation (LMs)

#### Idiosyncratic schemes

The following are the most successful LM models for each of the two performers (Table 2):

**Table 2.** Best idiosyncratic LM

Afzal Hussain: LM 1 (best)	
LM feature	Coefficients
*PitchminPre_rellog*	−1.65***
*Pitchmax_rellog*	+2.42***
VelocityMean	−1.28**
VelocitySD	+0.71.
HandDistanceMean_accord	+0.71*
*R^2^adj. (p < .001)*	***0.60***

Lakhan Lal Sahu: LM 2 (best)	
LM Feature	Coefficients
*PitchSD_abslog*	+0.56***
*Pitchmax_abslog*	+0.9***
HandDivergenceSD	+0.43*
OnsetAccelerationMean	+0.43*
*R^2^adj. (p < .001)*	***0.44***

According to LM 1 for vocalist Hussain, higher bodily effort levels are required for melodic glides that start from lower (*PitchminPre_rellog*) and ascend to higher (*Pitchmax_rellog*) degrees of the scale (the upper part of each octave) and are accompanied by hand movements that are slower (VelocityMean) but exhibit a larger speed variation (VelocitySD) while moving the hands further away from the point of reference (HandDistanceMean_accord). They conform to the descending character of rāga mode Jaunpurī and the increased melodic tension of the characteristic …/*b*7\*b*6 melodic movement compared to other typical double-sloped pitch glides of the mode, which is associated which touching the highest and most unstable 7^th^ degree for Jaunpurī before descending and having its melodic tension resolved upon the 6^th^ or 5^th^ degree. Hence, it could be argued that a rāga mode cannot be conceived as a uniform space of body activation, but it comprises distinct pitch-effort regions of potential activity, most likely inviting interactions with elastic objects as will be seen in the gesture classification models.

According to LM 2 for vocalist Sahu, higher bodily effort levels seem to be required for larger melodic glides (*PitchSD_abslog*) that reach up to higher pitches (*Pitchmax_abslog*) and are accompanied by hand movements that exhibit a larger variation of hand divergence (HandDivergenceSD, that is, change of speed in moving the hands apart from or closer to each other), with a strong mean acceleration at onset (OnsetAccelerationMean). As pitches are expressed here in an absolute logarithmic scale, they better reflect a strong association between voice and bodily effort that is rooted in the mechanical requirements of voice production (higher for very high and far low pitches) and may further confirm associations with the macro-organization of the ālāp improvisation with its progressively ascending pitches toward the climax.

**Figure 5.**
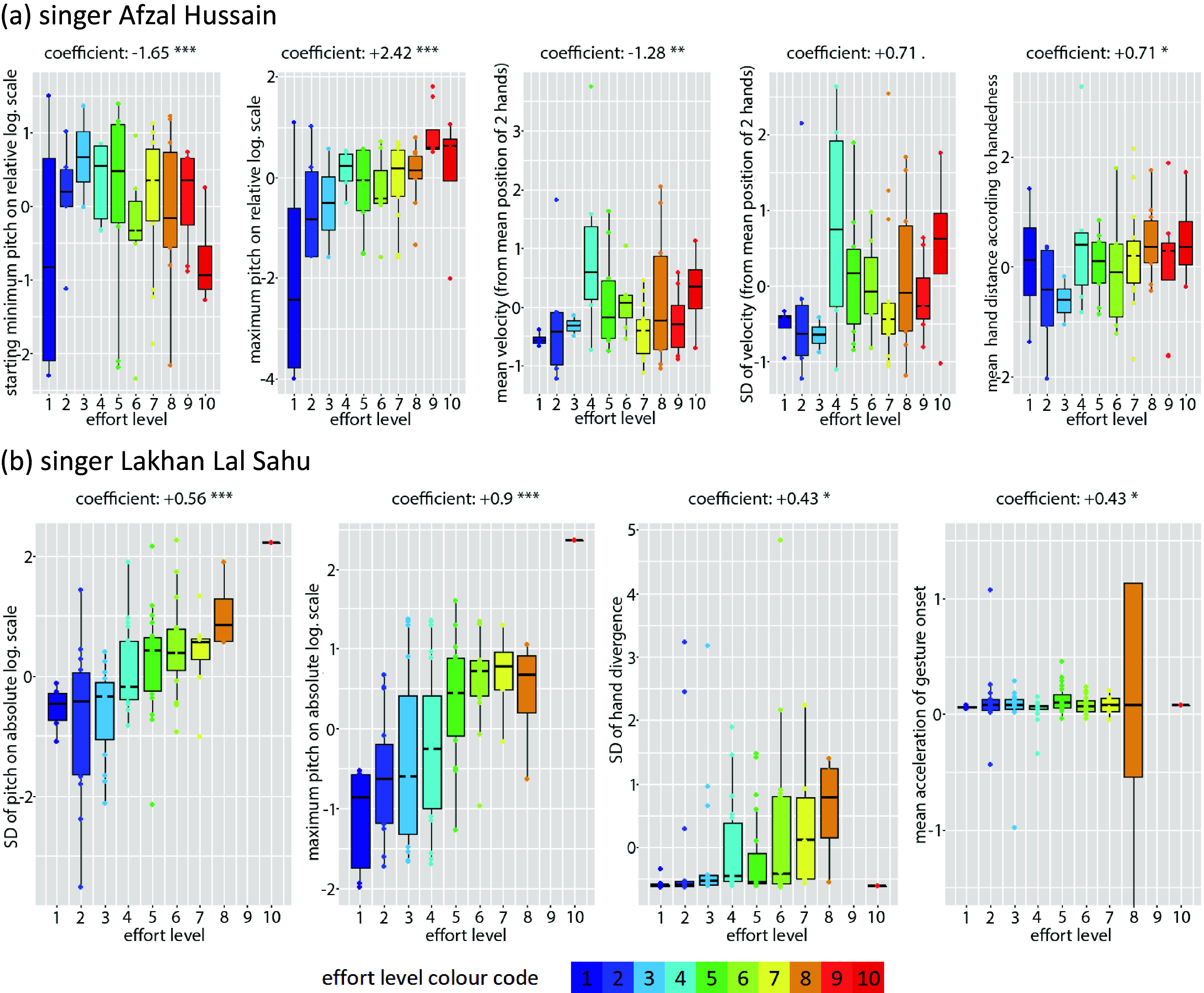
Boxplots for best (idiosyncratic) models LM 1 and 2 for singers Hussain (above) and Sahu (bottom), respectively, displaying the positive or negative correlation between each feature and the effort level, as well as the degree of confusion in the data distribution across effort level values (color coded for 1–10).

Despite some confusion in the distribution of the data across the effort level values, especially for movement features, overall association trends can be clearly visually discerned in Figure 5.

In summary, the above results indicate that in spite of the differences between the two idiosyncratic models, there is some degree of overlap in melodic qualities (size of melodic movement and duration of ascent), while movement features are quite different between the two performers. For instance, on a micro-timescale most melodic movements are performed within the boundaries of a single octave, which means that the models will often coincide in terms of melodic interval despite there being different features through which this is expressed. This is also supported by the higher probability values of the acoustic features compared to those of the movement features, which indicate that they are more significant for the estimation of effort. In fact, by keeping only the acoustic features of each, effort variance of more than half for vocalist Hussain (53%) and almost 40% for Sahu can be explained. However, cross-fitting models gave extremely poor results (40% for vocalist Hussain and 12% for Sahu).

#### Generic cross-performer schemes

Largely overlapping models (apart from one movement feature) of a lower, but still relatively good, fit were also produced with the same direction of contribution and similar statistical significance by each feature (Table 3).

**Table 3.** Most overlapping LM

LM similar	Afzal Hussain: LM 3 (similar)	Lakhan Lal Sahu: LM 4 (similar)
Feature	Coefficients	Coefficients
*PitchminPre_abslog*	−2.13***	−0.95***
*Pitchmax_abslog*	+2.3***	+1.91***
VelocityMean	−1.34**	−1.31**
VelocitySD	+0.93*	+1.35**
HandDistanceMean_accord	+0.65*	–
*R^2^adj. (p < .001)*	***0.53***	***0.42***

According to LM 3 and LM 4, higher bodily effort levels are required by both singers for melodic movements that start from a lower (*PitchminPre_abslog*) and reach up to a higher (*Pitchmax_abslog*) pitch and are accompanied by movements which are slow on average (VelocityMean) but exhibit a larger speed variation (VelocitySD), and in the specific case of vocalist Hussain, while moving the hands further away from the point of reference (HandDistanceMean_accord). As pitch is expressed here on an absolute logarithmic scale, it better reflects the mechanical effort required for producing notes in the highest or lowest vocal range rather than some rules of melodic organization or mental aspects of effort.

**Figure 6.**
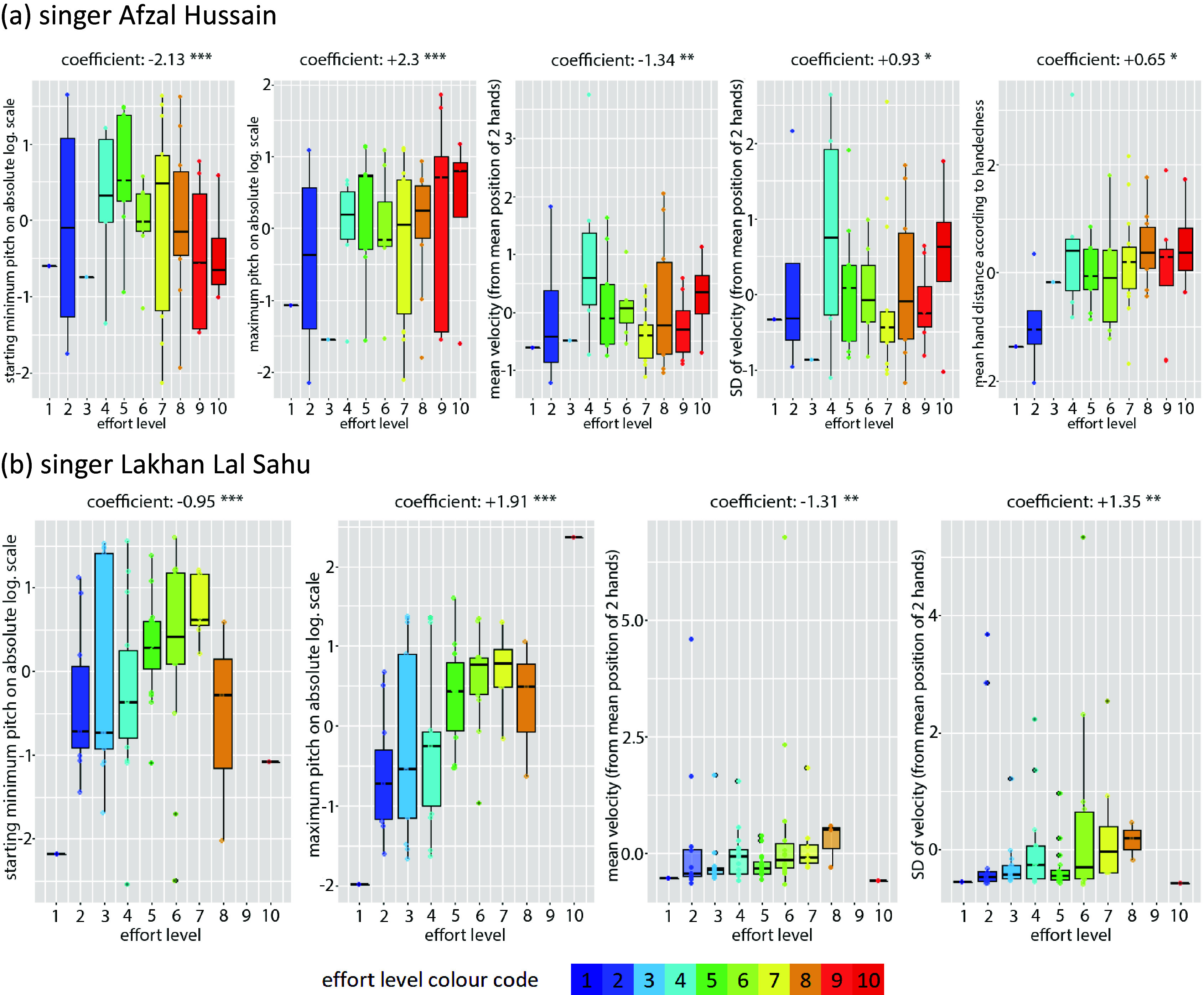
Boxplots for generic LM models 3 and 4 for singers Hussain (above) and Sahu (bottom), respectively, displaying the positive or negative correlation between each feature and the effort level, as well as the degree of confusion in the data distribution across effort level values (color coded for 1–10).

Again, the boxplots of Figure 6 illustrate the trends described by the models despite some confusion in the distributions, especially for mean and SD of velocity for singer Sahu.

It can be concluded that a relatively good estimation of effort level variance can be achieved by using almost the same combination of easily extracted global features. Most features are shared in terms of type, direction of contribution, and statistical significance, meaning that they contribute in similar ways to the estimation of effort. The lower goodness of fit of these models in comparison to the best selected models discussed in the previous section reflects their more generic power in describing MIIOs for both performers. In fact, effort variance of 46% for vocalist Hussain (53%) and 37% for Sahu can be achieved by keeping only the two acoustic features.

### Gesture Classification (GLMs)

#### Idiosyncratic schemes

The most successful GLM models in classifying interactions with rigid versus elastic objects are given in Table 4:

**Table 4.** Best idiosyncratic GLM in classifying interactions with rigid versus elastic objects

Afzal Hussain: GLM 1 (best)		
GLM Features	Coefficients	AUC
*timemax*	−3.04**	0.84
*Pitchmax_rellog*	−2.93**	0.72
*PitchSD_abslog*	−1.17	0.63
AccelerationMean	+3.4*	0.54
HandDivergenceSD	−3.12**	0.61
*AUC*		***0.95***

Lakhan Lal Sahu: GLM 2 (best)		
GLM Features	Coefficients	AUC
*coeff_2_*	+0.71*	0.63
*PitchSD_abslog*	−0.73**	0.7
VelocityMean	−1.48*	0.65
HandDistanceMean	+0.78*	0.54
*AUC*		***0.8***

According to GLM 1 for vocalist Hussain, interactions with elastic objects are more likely associated with slower (*timemax*) and larger (*PitchSD_abslog*) melodic movements that ascend to a higher degree of the scale (*Pitchmax_rellog*, i.e., upper part of the octave) and are performed by hand gestures that exhibit a lower variation in speed (AccelerationMean, computed on the average position of the hands) and a larger variation in the hands’ divergence (HandDivergenceSD, the speed in moving the hands apart). Results conform well to the descending character of rāga mode Jaunpurī, in which target notes are not approached directly in ascent but always through a higher note by which they are first attracted. As the 7^th^ is the highest and most unstable scale degree in this mode, it naturally forces a change of direction in pitch movement (ascent vs. descent) that can be paralleled to the change of direction in deforming an elastic object caused by the opposing force progressively increasing in proportion to displacement. It could therefore be suggested that the MIIO type for Hussain is linked to the voice based on the grammatical rules of the rāga mode and shared cross-modal morphologies. It could be argued that imagined opposing forces reflect the qualities of melodic movement; these imagined forces are not arbitrary and are not of the same nature over the entire pitch space, but they serve the potential needs of melodic expression and the raga structure, which makes the performer switch between object types and gesture classes according to the interactions that these afford (stretching: recoil force, pulling: gravitation or friction).

According to GLM 2 for vocalist Sahu, interactions with elastic objects are more likely to be performed with longer (time) and larger (pitch interval) melodic movements (*coeff_2_*, *PitchSD_abslog*) and with the hands moving faster (VelocityMean) but remaining bound to each other (HandDistanceMean), with all pitches being expressed here—as in LM 2 for this singer—on an absolute logarithmic scale which is independent of the rāga mode scale.

**Figure 7.**
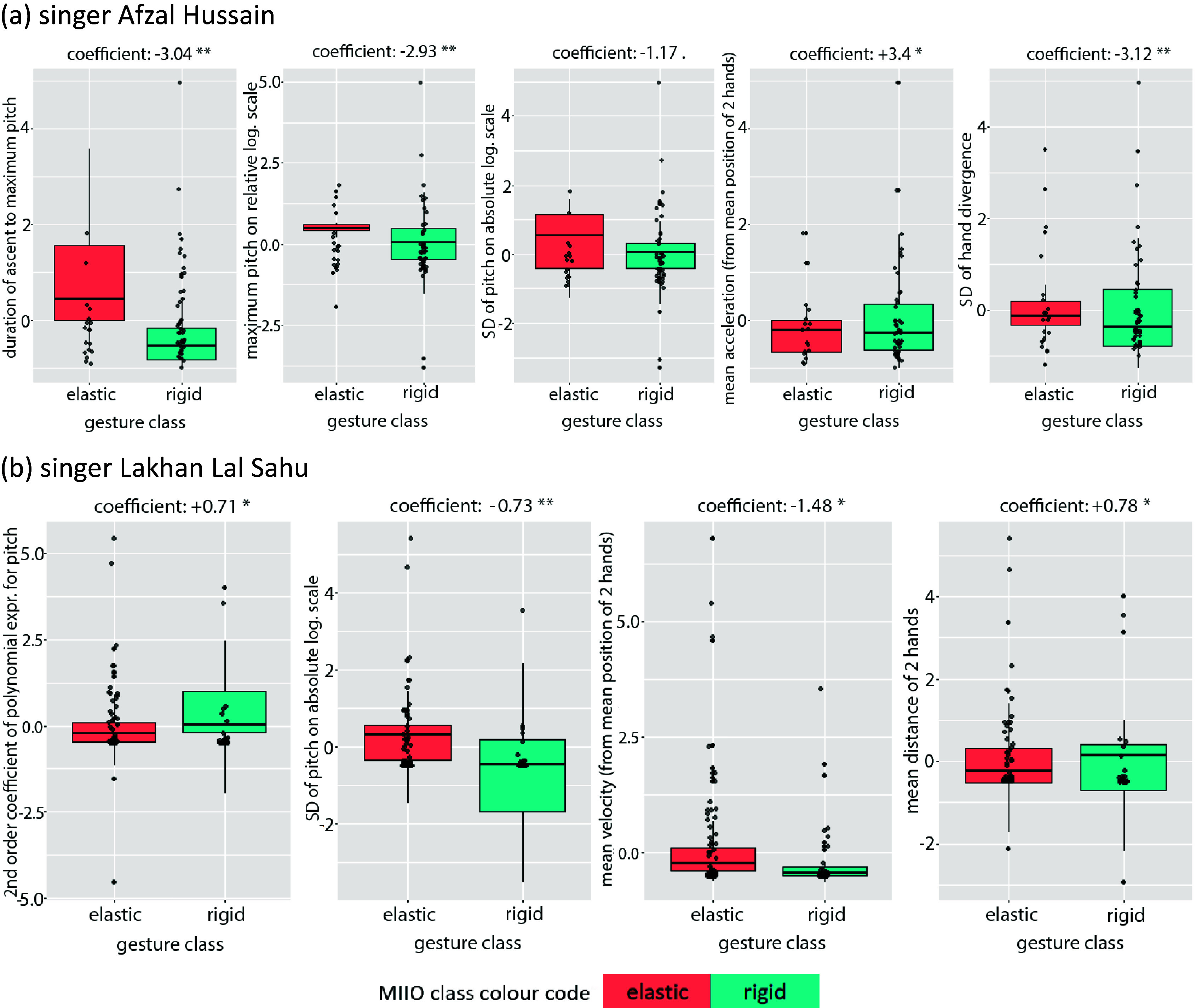
Boxplots for best (idiosyncratic) GLM models 1 and 2 for vocalists Hussain (above) and Sahu (bottom), respectively, displaying the positive or negative correlation between each feature and the gesture classes, as well as the degree of confusion in the data distribution across classes (color coded for elastic vs. rigid).

The boxplots of Figure 7 display the distributions of individual features. Despite some contradictions, the median values are in line with the trends described by the models.

In summary, there is a certain degree of overlap in the melodic qualities of the best GLM models (interval and duration of melodic movement), but movement features are different. This possibly highlights the idiosyncratic factor in performing MIIOs, which also renders the task of describing them by a small number of movement features non-trivial.

#### Generic cross-performer schemes

Although classification success rate may not be as high, the following are the two most overlapping GLM models across performers (Table 5):

**Table 5.** Most overlapping GLM in classifying interactions with rigid versus elastic objects

GLM	Afzal Hussain: GLM 3 (similar)		Lakhan Lal Sahu: GLM 4 (similar)	
Features	Coefficients	AUC	Coefficients	AUC
*PitchMean_abslog*	+1.45**	0.59	+0.51	0.51
*PitchSD_abslog*	−1.74**	0.63	−1.03***	0.7
HandDistanceMean	−1.1**	0.63	+0.98**	0.54
VelocityMean	–	–	−1.58*	0.65
*Overall*		***0.86***		***0.78***

**Figure 8.**
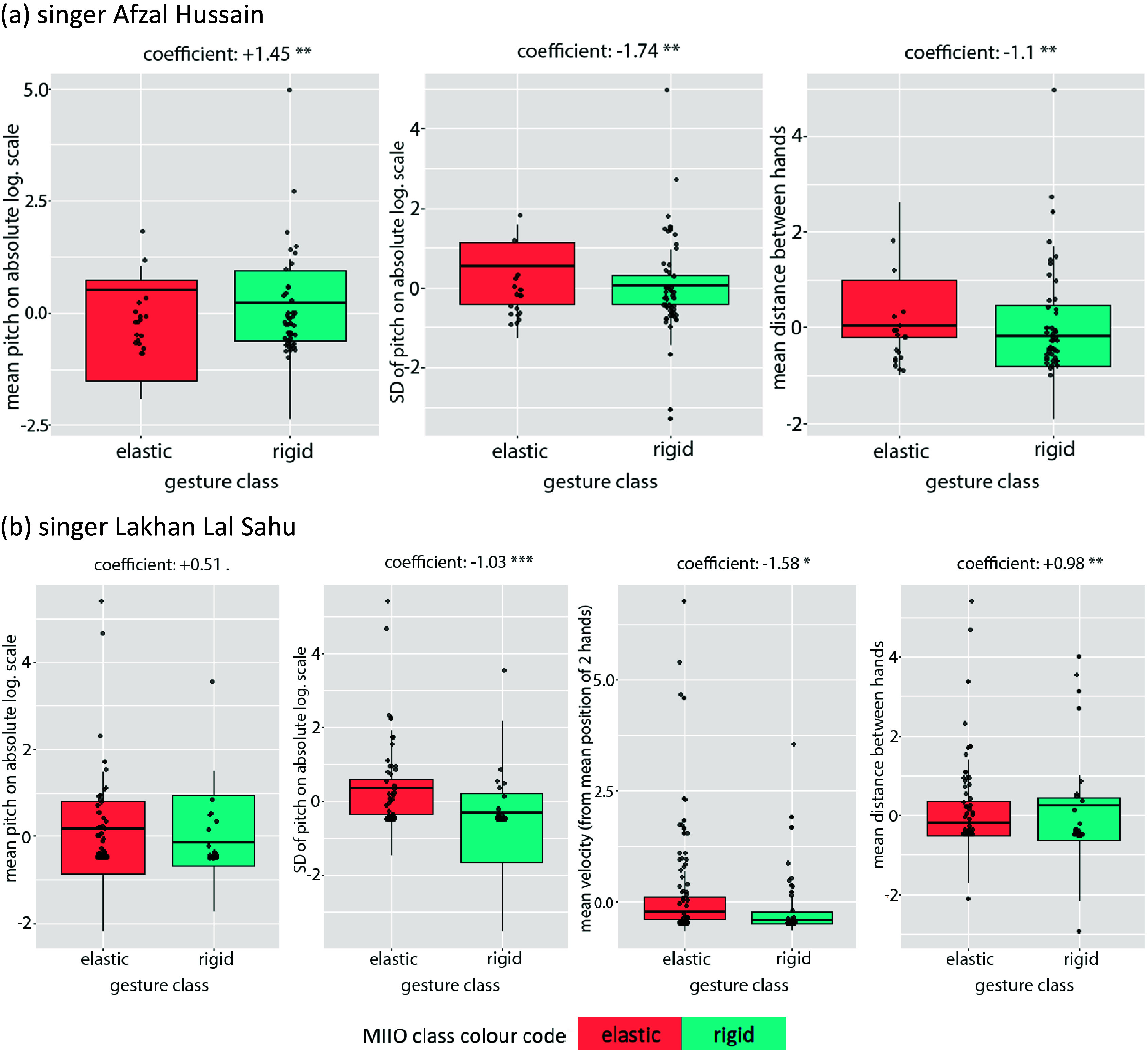
Boxplots for generic GLM models 3 and 4 for vocalists Hussain (above) and Sahu (bottom), respectively, displaying the positive or negative correlation between each feature and the gesture classes, as well as the degree of confusion in the data distribution across classes (color coded for elastic vs. rigid).

According to GLM 3 and 4, interactions with elastic objects are more likely to be performed with larger (*PitchSD_abslog*) melodic movements at lower pitches (*PitchMean_abslog*, on an absolute logarithmic scale) and with the hands moving further apart (HandDistanceMean) for vocalist Hussain and approaching each other but faster (HandDistanceMean, VelocityMean) in the case of vocalist Sahu. Similarly to the most overlapping LMs (3,4), pitch is again expressed on an absolute logarithmic scale, hence better reflecting the mechanical effort required for producing notes. It is interesting that just the use of a single (*PitchMean_abslog*) melodic feature yields a significant classification rate (0.63 and 0.7 AUC, respectively), showing how important this feature is. The boxplots of Figure 8 display the distribution of feature values, which—despite some confusion observed in the median values, mostly for the mean pitch by vocalist Sahu—coincides with the trends described by the models.

Again here, there is a certain overlap between the two models in acoustic features, but this is not so for movement features; even the single movement feature that is shared contributes in opposite directions. The difference in movement qualities illustrates alternative ways of interacting with an object, which depend on the balance of power between the performer’s actions and the opposing resistance acted by the imagined object (stiffness in stretching an elastic object or weight in moving a rigid object in space). Vocalist Hussain seems to be more effective in imagining defying the opposing forces, while Sahu seems to be mostly giving in. Additionally, there are differences in the statistical significance—and therefore the relative contribution—of individual (acoustic) features on the models, with the interval of the melodic movement being the most important (larger for interactions with elastic objects).

## Conclusion

The motivation for this work was twofold: On the one hand, to gain a deeper understanding of the role of effort during MIIOs in Dhrupad singing practice and on the other hand—based on this acquired knowledge—to devise formalized descriptions of effort for gestures that are inspired by familiar interactions with the real world, which could lead to the development of more intuitive gesture-sound mappings and physically plausible sounds in EMIs. The study thus reports the first ecologically valid mapping of sound sculpting gestures and their physical characteristics in respect to effort, which was achieved by applying a combination of qualitative and quantitative methods to original recordings of performances. Based on a small number of statistically significant movement and acoustic features it has been possible to develop compact LMs of a reasonably good fit for effort estimation and gesture classification and by this to reject the null hypothesis that effort and MIIO types are unrelated to the melodic content. Results demonstrate a higher degree of cross-performer overlap and a higher statistical significance for acoustic than for movement features, which suggests either different idiosyncratic schemes of movement or the limited success of the chosen low-level movement features in capturing the individual nuances of the way these gestures are performed by each vocalist.

These observations may lead to the conclusion that there is a significant association between effort and melodic qualities, but the way this is displayed by the performers’ hands is less obvious, more idiosyncratic, and perhaps also less consistent. Different cross-modal schemes were revealed for the performers analyzed. For vocalist Hussain, the melodic qualities mostly refer to pitch features that reflect the melodic organization of the rāga mode on a meso-level as pitch glides (pitch in LM 1 and GLM 1 is calculated on a relative logarithmic scale), thus primarily mental and conceptual aspects, while for Sahu they mostly refer to absolute pitch values that reflect the biomechanical requirements of voice production and the macro-structure of the improvisation (pitch in LM 2 and GLM 2 is calculated on an absolute logarithmic scale), thus both mental and bodily aspects. This also confirms results from the qualitative analysis and justifies a double grounding in the role of effort in gesture-sound associations—both a biomechanical (strain of vocalization) and a cognitive (organization of melodic material and conceptual cross-modal morphological analogies)—while performers may also switch between or combine these two modes of engagement. The mechanical requirements and the morphological analogies seem to have a more generic power, as they are shared between the two singers in LM and GLM 3, 4.

Hence, MIIOs offer a special case where motor imagery is “materialized” through effortful physical actions directed toward an imagined object. It could be suggested that the musicians’ capacity of imagining musical sound is facilitated through the retrieval of motor programs and image schemata from well-known interactions with real objects and that this may be exactly the reason for which imaginary objects are employed (bearing similarities to Godøy’s (2009) model of the gesture-sonorous object). Specifically, MIIOs can serve the potential needs of melodic expression by affording a dual facilitating role; either mechanical with respect to the bodily power required for producing the sound or mental in highlighting potential regions of special activity in the melodic organization, which is achieved by imagining opposing forces of a different nature and magnitude in different pitch regions and conveying the required effort thereof. Despite the flexibility in the way Dhrupad vocalists might use their hands in illustrating embodied concepts while singing, there is ample evidence of more generic associations between classes of MIIOs, their exerted effort levels and melody that are not necessarily performer-specific or stylistic and provide good evidence for non-arbitrariness.

These findings lead to suggesting that the MIIO metaphor can play a facilitating role in EMIs too by helping performers effectively control digital sounds through the retrieval of robust motor programs. By integrating the dynamic properties of effort and linking them to melodic qualities through gesture-sound mappings that conform to the formalized descriptions of the results, the expressive power of EMIs can be enhanced. This study has primarily revealed links between effort and pitch-related features; however, as image schemata (of MIIOs) represent extracted versions of general features and qualities of sensorimotor experiences (Johnson, 1987), other musical aspects pertaining to the dynamic character of effort may be further revealed by extending this analysis to a larger dataset or other music genres. Existing examples of virtual musical instruments building on action-based metaphors similar to MIIOs could benefit from these mappings (Françoise, 2015), for instance, the “*SculpTon”* by Boem (2014), the “*DAMPER”* by Bennett et al. (2007), and the original concept of “*Sound Sculpting”* by Mulder (1998).

In case of using the more generic LM 3 (Table 3) for mapping acoustic and movement features to effort in these instruments, the analytic description would be:


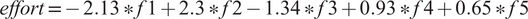
 , where:

f1 = starting minimum pitch on relative logarithmic scale (in relation to the tonic).

f2 = maximum pitch on absolute logarithmic scale (pitch height).

f3 = mean value of velocity calculated on the mean position of the two hands.

f4 = SD of velocity calculated on the mean position of the two hands.

[f5 = mean hand distance according to handedness, only used for singer Sahu].

**Figure 9.**
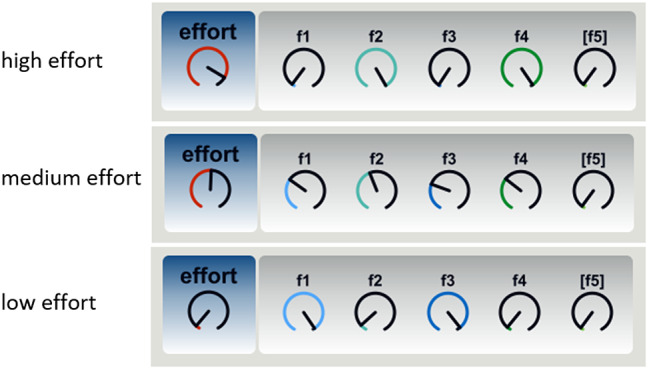
Schematic of mapping movement and acoustic features to high–medium–low effort levels according to the more generic model LM 3.

Figure 9 illustrates a schematic representation of parameter mapping for an EMI using LM 3, showing how acoustic and movement features would change when a user delivers less, medium, or high levels of effort.

Finally, the results of this study lead to further proposing that in designing an EMI a more flexible mapping scheme should be aimed for, which would enable the performer to switch between different mapping modes, reflecting effort-related concepts that are either conceptual or physical, and either idiosyncratic or more generic. Also, based on the amodal character of effort that was inferred from a combination of acoustic and movement features, it makes sense to propose a new hierarchical approach (Françoise et al., 2012) in which effort can be used as a perceptual mapping layer that merges rather than matches movement to sound as two parallel processes emerging from a common idea.

## Future Work and Implications to Wearable Technologies

Applying a combination of qualitative and quantitative methods to real performance recordings has brought about a difficult trade-off between an ethnomusicological approach and a systematic analysis of designed and repeatable experiments. Thus, as much as bringing the advantages of ecological validity, this approach has also posed important challenges and highlighted limitations that are worth discussing in the context of wearable technologies.

Τhe limited dataset—a result of the choice of ecological validity—models may be overfitting the actual data and thus larger datasets would be beneficial for enabling a more systematic comparison between performers, performances, and rāga modes and examining the generic power of current results, which can be also used in effort prediction tasks on new recorded material. Although starting from the assumption of linear links is a valid first step, identification of patters in the models’ residuals leads to the assumption that the exploration of non-linear elements might further improve the estimation of responses. Since the way an observer makes assessments on perceived effort levels remains a non-transparent process, it is also important to disambiguate observers’ criteria, specifically discriminating between the bodily (acoustic and movement) and the mental (imagery). This work should also be extended to examine the temporal congruence between sets of effort levels and time-varying (Maestre et al., 2006; Battey, 2004) rather than global measures, which better account for the dynamic profiles of gestures.

By acknowledging the dynamic properties of effort as innately linked to artistic expressive power (Luciani, 2009) and by better understanding its role in the relationships between movement and the singing voice in MIIOs, this work can contribute to enabling advances in cross-modal mappings and the expressiveness of EMIs. However, the manual annotation of effort levels, dictated by the absence of a widely accepted ground truth, could be criticized for subjectivity, and it is also not useful in developing real-time applications. Now that the close link between sound and effort has been ascertained, an interesting follow-up would be to explore suitable quantifiable measures and sensor candidates for directly capturing effort in driving synthesized sounds, such as in “sound sculpting.” Physiological measures and capture devices, such as electromyography or electroencephalography, seem to hold good promise (Tanaka & Ortiz, 2017) and if proven suited they could offer a successful way to incorporate effort in real-time audio applications. However, considering the complex—perceptual and multimodal—nature of effort, in being subjective and consisting of both overt and covert aspects, this is not a trivial task and therefore systematic work needs to be done in this direction.

## Data Availability

Audio-visual materials of the two performances used in this paper are available at: https://collections.durham.ac.uk/files/r10p096691p#.YOROyegzYdU and https://collections.durham.ac.uk/files/r12b88qc178#.YORO1OgzYdU. Mocap data are not openly accessible but can be only made available upon request.

## Footnotes

1 The idea of the body as a mediation between mind and matter has been criticised by Schiavio (2014) as an unintentional upholding of the Cartesian body–mind dichotomy.

2 Although some of the language used throughout this paper (e.g., when referring to the “physical” and “mental” aspects of effort) risks implying that it takes a constructivist stance, and it clearly subscribes to theories of embodied (music) cognition (Varela et al., 1991). The choice of language was deliberate and aims at drawing attention to the understanding of effort as something that carries both overt (physical, i.e., can be seen and heard) and covert (conceptual/mental, reflecting cognitive strain) aspects. Alternative terms with a clear focus on embodiment were considered less intuitive and counterproductive in the context of a journal with a focus on wearable technologies.

3 That is, the “space between the notes,” which is often considered in Dhrupad more important than the discrete notes themselves (Van der Meer & Rao, 2006).

4 Pitch was extracted semi-automatically through autocorrelation in Praat due to the high robustness of the algorithm in detecting the pitch of the voice accurately in the ambience of drone instrument(s).

5 Following R-symbols are used to visually specify level of significance: “***” for 0≤ *p*<0.001, “**” for 0.001 ≤ *p*<0.01, “*” for 0.01≤*p*<0.05, “.” for 0.05 ≤ *p*<0.1, “ ” for 0.1 ≤ *p*<1.

## Appendix A: Feature Name Abbreviations

**Table A1.** Abbreviation of all features that featured in the successful models presented in the analysis

Feature	Suffix	Scale
*PitchminPre*	*_abslog*	Starting minimum pitch of melodic glide calculated on an Absolute or relative logarithmic scale (not for descending glides)
	*_rellog*	
*Pitchmax*	*_abslog*	Maximum pitch of melodic glide calculated on an absolute or relative logarithmic scale
	*_rellog*	
*PitchSD*	*_abslog*	Standard deviation of fundamental frequency for a melodic glide calculated on an absolute logarithmic scale
*PitchMean*	*_abslog*	Mean fundamental frequency for a melodic glide calculated on an absolute logarithmic scale
*timemax*		Timestamp of *Pitchmax* from beginning of melodic glide
*coeff_2_*		Second-order coefficient of polynomial expression for pitch contour:
*VelocityMean*		Mean value of velocity calculated on the mean three-dimensional position of the two hands
*VelocitySD*		Standard deviation of velocity calculated on the mean three-dimensional position of the two hands
*HandDistanceMean*		Mean distance of hands calculated between the two hands (no suffix) or according to handedness
	*_accord*	
*HandDivergenceSD*		Standard deviation of hand divergence calculated as 1st derivative of HandDistanceMean
*AccelerationMean*		Mean value of acceleration over time, calculated on the mean three-dimensional position of the two hands
*OnsetAccelerationMean*		Mean of acceleration calculated on the gesture onset as the second derivative of HandDistanceMean (15% of total duration for events of duration > 1.7 sec and 45% for events with duration ≤ 1.7 s)

## References

[r154] Alaoui SF, Caramiaux B, Serrano M and Bevilacqua F (2012) Movement qualities as interaction modality. Proceedings of the Designing Interactive Systems Conference on - DIS ’12. 10.1145/2317956.2318071

[r1] Autrimncpa (2012a) Jaunpuri. Retrieved from https://autrimncpa.wordpress.com/jaunpuri/ (last accessed 5 July 2021).

[r2] Autrimncpa (2012b) Malkauns. Retrieved from https://autrimncpa.wordpress.com/malkauns/ (last accessed 5 July 2021).

[r3] Bartenieff I and Lewis D (1980) Body Movement: Coping with the Environment. New York: Routledge.

[r4] Battey B (2004) Bézier spline Modeling of pitch-continuous melodic expression and ornamentation. Computer Music Journal 28(4), 25–39.

[r5] Battey B, Giannoukakis M and Picinali L (2015) Haptic control of multistate generative music systems. In International Computer Music Conference (ICMC). Denton, TX, USA.

[r6] Bennett P, Ward N, O’Modhrain S and Rebelo P (2007) DAMPER: A platform for effortful interface development. In Proceedings of the 7th International Conference on New Interfaces for Musical Expression. New York: Association for Computing Machinery, pp. 273–276.

[r7] Berdahl E, Niemeyer G and Smith III, JO (2009) Using haptics to assist Performers in making gestures to a musical instrument. In Proceedings of the 2009 International Conference on New Interfaces for Musical Expression, NIME 2009, Pittsburgh, PA, USA. New York: Association for Computing Machinery, pp. 177–182.

[r137] Bernhardt D and Robinson P (s. d.) Detecting Affect from Non-stylised Body Motions. Lecture Notes in Computer Science, 59–70. 10.1007/978-3-540-74889-2_6

[r8] Bevilacqua F, Schnell N, Françoise J, Boyer EO, Schwarz D and Caramiaux B (2017) Designing action–sound metaphors using motion sensing and descriptor-based synthesis of recorded sound materials. In Lesaffre M, Maes P-J and Leman M (eds), The Routledge Companion to Embodied Music Interaction. New York: Taylor & Francis, pp. 391–401.

[r9] Bigand E and Parncutt R (1999) Perceiving musical tension in long chord sequences. Psychological Research: An International Journal of Perception, Attention, Memory and Action 62(4), 237–254. 10.1007/s004260050053 10652864

[r10] Boem A (2014) Sculpton: A malleable tangible interface for sound sculpting. In Georgaki A and Kouroupetroglou G (eds), Proceedings of the 2014 Joint Conference: 40th International Computer Music Conference (ICMC) and 11th Sound and Music Computing Conference (SMC). Athens, Greece, pp. 747–743.

[r11] Broughton MC and Davidson JW (2014) Action and familiarity effects on self and other expert musicians’ Laban effort-shape analyses of expressive bodily behaviors in instrumental music performance: A case study approach. Frontiers in Psychology 5, 1201. 25400601 10.3389/fpsyg.2014.01201 PMC4212616

[r12] Brunkan MC (2015) The effects of three singer gestures on acoustic and perceptual measures of solo singing. International Journal of Music and Performing Arts 3(1), 35–45.

[r13] Bruya B and Tang YY (2018) Is attention really effort? Revisiting Daniel Kahneman’s influential 1973 book attention and effort. Frontiers in Psychology 9, 1133. 30237773 10.3389/fpsyg.2018.01133 PMC6136270

[r14] Cambridge University Press (n.d.) Effort. In Cambridge *Business English Dictionary*. Retrieved from https://dictionary.cambridge.org/dictionary/english/effort (last accessed 2 February 2022).

[r15] Camurri A, Canepa C, Ghisio S and Volpe G (2009) Automatic classification of expressive hand gestures on tangible acoustic interfaces according to Laban’s theory of effort. Lecture Notes in Computer Science 5085, 151–162.

[r16] Camurri A, De Poli G, Leman M and Volpe G (2001) A multi-layered conceptual framework for expressive gesture applications. In Proceedings of the Workshop on Current Research Directions in Computer Music, Barcelona, Spain, pp. 29–34.

[r17] Camurri A, Lagerlöf I and Volpe G (2003) Recognizing emotion from dance movement: Comparison of spectator recognition and automated techniques. International Journal of Human Computer Studies 59(1–2), 213–225.

[r18] Camurri A and Trocca R (2000) Movement and gesture in intelligent interactive music systems. In Wanderley M and Battier M (eds), Trends in Gestural Control of Music (CDROM). Paris: Ircam/Centre Pompidou.

[r19] Caramiaux B, Bevilacqua F, Schnell N (2009) Towards a gesture-sound cross-modal analysis. In Kopp S and Wachsmuth I (eds), Gesture in Embodied Communication and Human-Computer Interaction. Lecture Notes in Computer Science, vol 5934. Berlin–Heidelberg: Springer, pp. 158–170.

[r136] Caridakis G, Raouzaiou A, Bevaequa E, Maneini M, Karpouzis K, Malatesta L and Pelachaud C (2007) Virtual agent multimodal mimicry of humans. Language Resources and Evaluation 41(3–4), 367–388. 10.1007/s10579-007-9057-1

[r20] Castagné N and Cadoz C (2005) A goals-based review of physical modelling. In Proceedings of the 2005 International Computer Music Conference, Barcelona, Spain, pp. 343–346.

[r21] Castagné N, Cadoz C, Florens JL and Luciani A (2004) Haptics in computer music: A paradigm shift. In Proceedings of Eurohaptics 2004, Munich, Germany (June 5-7, 2004), pp. 422–425.

[r135] Castellano G and Maneini M (2009) Analysis of Emotional Gestures for the Generation of Expressive Copying Behaviour in an Embodied Agent. Gesture-Based Human-Computer Interaction and Simulation, 193–198. 10.1007/978-3-540-92865-2_21

[r22] Cheval B and Boisgontier MP (2021) The theory of effort minimization in physical activity. Exercise and Sport Sciences Reviews 49(3), 168. 34112744 10.1249/JES.0000000000000252 PMC8191473

[r23] Chi D, Costa M, Zhao L and Badler N (2000) The EMOTE model for effort and shape. In Proceedings of the 27th ACM SIGGRAPH Conference on Computer Graphics and Interactive Techniques, New Orleans, Louisiana, USA. New York: ACM Press/Addison-Wesley Publishing Co., pp.173–182.

[r24] Clarke EF (2005) Ways of Listening: An Ecological Approach to the Perception of Musical Meaning. Oxford: Oxford University Press.

[r25] Clayton M and Leante L (2013) Embodiment in music performance. In Clayton M, Dueck B and Leante L (eds), Experience and Meaning in Music Performance. Oxford: Oxford University Press, pp. 188–207.

[r26] Cox A (2011) Embodying music: Principles of the mimetic hypothesis. Music Theory Online 17(2).

[r27] Cox A (2016) Music and Embodied Cognition: Listening, Moving, Feeling, and Thinking. Bloomington: Indiana University Press.

[r28] d’Escriván J (2006) To sing the body electric: Instruments and effort in the performance of electronic music. Contemporary Music Review 25(1), 183–191.

[r29] Dewey J (1897) The psychology of effort. The Philosophical Review 6(1), 43–56.

[r30] dos Santos LCGF (2013) *Laban Movement Analysis: A Bayesian Computational Approach to Hierarchical Motion Analysis and Learning.* Doctoral thesis, Universidade de Coimbra, Coimbra, Portugal. Retrieved from https://estudogeral.sib.uc.pt/jspui/bitstream/10316/24291/1/Laban%20Movement%20Analysis.pdf (last accessed 5 July 2021).

[r31] Eitan Z and Granot RY (2006) How music moves. Music Perception 23(3), 221–248.

[r32] Enoka RM and Stuart DG (1992) Neurobiology of muscle fatigue. Journal of Applied Physiology 72(5), 1631–1648. 1601767 10.1152/jappl.1992.72.5.1631

[r33] Essl G and O’Modhrain S (2006) An enactive approach to the design of new tangible musical instruments. Organised Sound 11(3), 285–296.

[r34] Fatone GA, Clayton M, Leante L and Rahaim M (2011) Imagery, melody and gesture in cross-cultural perspective. In Gritten A and King EF (eds), New Perspectives on Music and Gesture. Surrey: Ashgate, pp. 203–220.

[r35] Fdili Alaoui S, Françoise J, Schiphorst T, Studd K and Bevilacqua F (2017) Seeing, sensing and recognizing Laban movement qualities. In Proceedings of the 2017 CHI Conference on Human Factors in Computing Systems. New York: Association for Computing Machinery, pp. 4009–4020.

[r36] Fels S, Gadd A and Mulder A (2002) Mapping transparency through metaphor: Towards more expressive musical instruments. Organised Sound 7(2), 109–126.

[r37] Françoise J (2015) *Motion-Sound Mapping by Demonstration.* Doctoral thesis, Université Pierre et Marie Curie, Ircam, Paris, France. Retrieved from https://tel.archives-ouvertes.fr/tel-01161965/document (last accessed 5 July 2021).

[r38] Françoise J, Caramiaux B and Bevilacqua F (2012) A hierarchical approach for the design of gesture-to-sound mappings. In Proceedings of the 9th Sound and Music Computing Conference, Copenhagen, Denmark, pp. 233–240.

[r39] Françoise J, Fdili Alaoui S, Schiphorst T and Bevilacqua F (2014). Vocalizing dance movement for interactive sonification of laban effort factors. In Proceedings of the 2014 Conference on Designing Interactive Systems. New York: Association for Computing Machinery, pp. 1079–1082.

[r40] Freed DJ (1990) Auditory correlates of perceived mallet hardness for a set of recorded percussive sound events. The Journal of the Acoustical Society of America 87(1), 311–322. 2299041 10.1121/1.399298

[r41] Garnett GE and Goudeseune C (1999) Performance factors in control of high-dimensional space. In Proceedings of the 1999 International Computer Music Conference (ICMC), Beijing, China, October 22-27, 1999. Michigan: Michigan Publishing.

[r42] Gibet S (2010) Sensorimotor control of sound-producing gestures. In Leman M and Godøy RI (eds), Musical Gestures: Sound, Movement, and Meaning. New York: Routledge, pp. 212–237.

[r43] Gibson J (1977) The theory of affordances. In Shaw R and Bransford J (eds), Perceiving, Acting, and Knowing: Toward an Ecological Psychology. Hillsdale, NJ: Lawrence Erlbaum, pp. 67–82.

[r44] Glowinski D, Coll SY, Baron N, Sanchez M, Schaerlaeken S and Grandjean D (2017) Body, space, and emotion: A perceptual study. Human Technology: An Interdisciplinary Journal on Humans in ICT Environments 13, 32–57.

[r45] Godøy RI (2009) Geometry and effort in gestural renderings of musical sound. In Sales Dias M, Gibet S, Wanderley MM and Bastos R (eds), Gesture-Based Human-Computer Interaction and Simulation. Lecture Notes in Computer Science, vol 5085. Berlin–Heidelberg: Springer, pp. 205–215.

[r46] Granot RY and Eitan Z (2011) Musical tension and the interaction of dynamic auditory parameters. Music Perception 28(3), 219–246.

[r139] Hachimura K, Takashina K and Yoshimura M (s. d.) Analysis and evaluation of dancing movement based on LMA. ROMAN 2005. IEEE International Workshop on Robot and Human Interactive Communication, 2005. 10.1109/roman.2005.1513794

[r47] Hackney P (2002) Making Connections: Total Body Integration through Bartenieff Fundamentals. New York: Routledge.

[r48] Hartmann B, Mancini M, Buisine S and Pelachaud C (2005) Design and evaluation of expressive gesture synthesis for embodied conversational agents. In Proceedings of the 4th International Joint Conference on Autonomous Agents and Multi Agent Systems, Utrecht, The Netherlands, pp. 1095–1096.

[r50] Huron DB (2006) Sweet Anticipation: Music and the Psychology of Expectation. Cambridge, MA: MIT Press.

[r51] Inzlicht M, Shenhav A and Olivola CY (2018) The effort paradox: Effort is both costly and valued. Trends in Cognitive Sciences 22(4), 337–349. 29477776 10.1016/j.tics.2018.01.007 PMC6172040

[r52] Jagiello R, Pomper U, Yoneya M, Zhao S and Chait M (2019) Rapid brain responses to familiar vs. unfamiliar music—An EEG and pupillometry study. Scientific Reports 9(1), 1–13. 31666553 10.1038/s41598-019-51759-9 PMC6821741

[r53] James G, Witten D, Hastie T and Tibshirani R (2013) An Introduction to Statistical Learning, Vol. 6. New York: Springer.

[r54] Jensenius AR (2007) Action-Sound: Developing Methods and Tools to Study Music-Related Body Movement. Doctoral thesis, University of Oslo, Oslo, Norway. Retrieved from https://www.duo.uio.no/handle/10852/27149 (last accessed 5 July 2021).

[r55] Johnson M (1987) The Body in the Mind: The Bodily Basis of Meaning, Imagination, and Reason. Chicago: University of Chicago Press.

[r56] Kahneman D (1973) Attention and Effort, Vol. 1063. Englewood Cliffs, NJ: Prentice-Hall.

[r57] Kahneman D and Beatty J (1967) Pupillary responses in a pitch-discrimination task. Perception & Psychophysics 2(3), 101–105.

[r133] Kapadia M, Chiang I, Thomas T, Badler NI and Kider JT (2013) Efficient motion retrieval in large motion databases. Proceedings of the ACM SIGGRAPH Symposium on Interactive 3D Graphics and Games - I3D ’13. 10.1145/2448196.2448199

[r58] Krefeld V and Waisvisz M (1990) The hand in the web: An interview with Michel Waisvisz. Computer Music Journal 14(2), 28–33.

[r59] Krueger J (2014) Affordances and the musically extended mind. Frontiers in Psychology 4, 1003. 24432008 10.3389/fpsyg.2013.01003 PMC3880934

[r60] Kurth E (1922) Grundlagen Des Linearen Kontrapunkts: Bachs Melodische Polyphonie. Berlin: M. Hesse.

[r61] Küssner M (2014) Shape, Drawing and Gesture: Cross-Modal Mappings of Sound and Music. Doctoral thesis, King’s College London, University of London, London, UK. Retrieved from https://kclpure.kcl.ac.uk/portal/en/theses/shape-drawing-and-gesture(2b4cf820-956f-4da9-a29e-1053870d656b).html (last accessed 5 July 2021).

[r62] Laban R and Lawrence FC (1974) Effort: Economy of Body Movement. Boston: Plays, Inc.

[r63] Laban R and Ullmann L (1971) The Mastery of Movement. Boston: Plays, Inc.

[r64] Lahav A, Saltzman E and Schlaug G (2007) Action representation of sound: Audiomotor recognition network while listening to newly acquired actions. The Journal of Neuroscience: The Official Journal of the Society for Neuroscience 27(2), 308–314. 17215391 10.1523/JNEUROSCI.4822-06.2007 PMC6672064

[r65] Leante L (2009) The lotus and the King: Imagery, gesture and meaning in a Hindustani Rāg. Ethnomusicology Forum 18(2), 185–206.

[r66] Leman M (2007) Embodied Music Cognition and Mediation Technology. Cambridge, MA: MIT press.

[r67] Leman M (2010) Music, gesture, and the formation of embodied meaning. In Leman M and Godøy RI (eds), Musical Gestures: Sound, Movement, and Meaning. New York: Routledge, pp. 126–153.

[r68] Leman M, Maes PJ, Nijs L and Van Dyck E (2018) What is embodied music cognition? In Springer Handbook of Systematic Musicology. Berlin–Heidelberg: Springer, pp. 747–760.

[r69] Lerdahl F and Krumhansl CL (2007) Modeling tonal tension. Music Perception 24(4), 329–366.

[r70] Luciani A, Florens JL, Couroussé D and Castet J (2009) Ergotic sounds: A new way to improve playability, believability and presence of virtual musical instruments. Journal of New Music Research 38(3), 309–323.

[r71] Luck G and Toiviainen P (2008) Exploring relationships between the kinematics of a singer’s body movement and the quality of their voice. Journal of Interdisciplinary Music Studies 2(1–2), 173–186.

[r72] Maes PJ, Dyck EV, Lesaffre M, Leman M and Kroonenberg PM (2014) The coupling of action and perception in musical meaning formation. Music Perception: An Interdisciplinary Journal 32(1), 67–84.

[r73] Maes PJ, Leman M, Lesaffre M, Demey M and Moelants D (2010) From expressive gesture to sound. Journal on Multimodal User Interfaces 3(1), 67–78.

[r74] Maestre E, Bonada J and Mayor O (2006) Modeling musical articulation gestures in singing voice performances. In Proceedings of the Audio Engineering Society - 121st Convention Papers 2006, San Francisco, California, vol. 1, pp. 471–479.

[r75] Maletic V (1987) Body, Space, Expression: The Development of Rudolf Laban’s Movement and Dance Concepts, Vol. 75. Berlin: Mouton de Gruyter.

[r76] Mana M (2007) Ustad Fariduddin Dagar. Flickr. Retrieved from https://www.flickr.com/photos/maitrajeevache/557558050/in/photostream/ (last accessed 5 July 2021).

[r77] Marcora S (2009) Perception of effort during exercise is independent of afferent feedback from skeletal muscles, heart, and lungs. Journal of Applied Physiology 106(6), 2060–2062. 18483166 10.1152/japplphysiol.90378.2008

[r78] Massin O (2017) Towards a definition of efforts. Motivation Science 3(3), 230

[r150] Mazzarino B, Peinado M, Boulie R, Volpe G and Wanderley MM (2009) Improving the Believability of Virtual Characters Using Qualitative Gesture Analysis. Gesture-Based Human-Computer Interaction and Simulation, 48–56. 10.1007/978-3-540-92865-2_5

[r79] McKenna VS and Stepp CE (2018) The relationship between acoustical and perceptual measures of vocal effort. The Journal of the Acoustical Society of America 144(3), 1643–1658. 30424674 10.1121/1.5055234 PMC6167228

[r80] Menin D and Schiavio A (2012) Rethinking musical affordances. Avant 3(2), 202–215.

[r151] Mentis HM and Johansson C (2013) Seeing movement qualities. Proceedings of the SIGCHI Conference on Human Factors in Computing Systems. 10.1145/2470654.2466462

[r82] Mion L, de Poli GD and Rapanà E (2010) Perceptual organization of affective and sensorial expressive intentions in music performance. ACM Transactions on Applied Perception 7(2), 1–21.

[r83] Moore CL and Yamamoto K (2012) Beyond Words: Movement Observation and Analysis. New York: Routledge.

[r84] Moore DS and McCabe GP (2006) Introduction to the Practice of Statistics, 5th Edn. New York: W. H. Freeman and Company.

[r85] Moran N (2007) *Measuring Musical Interaction: Analysing Communication in Embodied Musical Behaviour.* Doctoral thesis, Open University, Milton Keynes, UK. Retrieved from http://oro.open.ac.uk/39118/ (last accessed 5 July 2021).

[r153] Morita J, Nagai Y and Moritsu T (2013) Relations between body motion and emotion: Analysis based on Laban Movement Analysis. In Proceedings of the 35th Annual Meeting of the Cognitive Science Society, Berlin, Germany, pp. 1026–1031.

[r86] Mulder A (1998) *Design of Virtual Three-Dimensional Instruments for Sound Control.* Doctoral thesis, Rijks Universiteit Groningen, Groningen, The Netherlands. Retrieved from http://www.xspasm.com/x/sfu/vmi/AM98-thesis.pdf (last accessed 5 July 2021).

[r87] Mulder G (1986) The concept and measurement of mental effort. In: Hockey GRJ, Gaillard AWK and Coles MGH (eds), Energetics and Human Information Processing. NATO ASI Series, vol 31. Dordrecht: Springer, pp. 175–198.

[r140] Nakata T, Mori T and Sato T (2002) Analysis of Impression of Robot Bodily Expression. Journal of Robotics and Mechatronics, 14(1), 27–36. 10.20965/jrm.2002.p0027

[r88] Niewiadomski R, Mancini M and Piana S (2013) Human and virtual agent expressive gesture quality analysis and synthesis. In Rojc M and Campbell N (eds), Coverbal Synchrony in Human-Machine Interaction. Boca Raton, FL: CRC Press, pp. 269–292.

[r89] Nussbaum CO (2007) The Musical Representation: Meaning, Ontology, and Emotion. Cambridge, MA: MIT Press.

[r90] Nymoen K, Godøy RI, Jensenius AR and Torresen J (2013) Analyzing correspondence between sound objects and body motion. ACM Transactions on Applied Perception 10(2), 1–22.

[r91] O’Modhrain S and Gillespie RB (2018) Once more, with feeling: Revisiting the role of touch in performer-instrument interaction. In Papetti S and Saitis C (eds), Musical Haptics. Springer Series on Touch and Haptic Systems. Cham: Springer, pp. 11–27.

[r92] O’Regan JK and Noë A (2001) Acting out our sensory experience. Behavioral and Brain Sciences 24(5), 1011–1021.

[r93] O’Shea H and Moran A (2018) Are fast complex movements unimaginable? Pupillometric studies of motor imagery in expert piano playing. Journal of Motor Behavior 51, 371–384. 30277448 10.1080/00222895.2018.1485010

[r94] Olsen KN and Dean RT (2016) Does perceived exertion influence perceived affect in response to music? Investigating the “FEELA” hypothesis. Psychomusicology: Music, Mind, and Brain 26(3), 257.

[r95] Papadelis C, Kourtidou-Papadeli C, Bamidis P and Albani M (2007) Effects of imagery training on cognitive performance and use of physiological measures as an assessment tool of mental effort. Brain and Cognition 64(1), 74–85. 17335950 10.1016/j.bandc.2007.01.001

[r96] Paschalidou PS (2017) *Effort in Gestural Interactions with Imaginary Objects in Hindustani Dhrupad Vocal Music.* Doctoral dissertation, Durham University. Retrieved from http://etheses.dur.ac.uk/12308/ (last accessed 5 July 2021).

[r97] Paschalidou S and Clayton M (2015) Towards a sound-gesture analysis in Hindustani Dhrupad vocal music: Effort and raga space. Paper presented at *the (ICMEM) International Conference on the Multimodal Experience of Music*, Sheffield, UK, 23–25 March, 2015.

[r98] Paschalidou S, Eerola T and Clayton M (2016) Voice and movement as predictors of gesture types and physical effort in virtual object interactions of classical Indian singing. In Proceedings of the (MOCO) 3rd International Symposium on Movement and Computing, Thessaloniki, Greece, 5–6 July 2016. New York: Association for Computing Machinery, p. 45:1--45:2.

[r99] Pearson L (2016) *Gesture in Karnatak Music: Pedagogy and Musical Structure in South India.* Doctoral thesis, Durham University, Durham, UK. Retrieved from http://etheses.dur.ac.uk/11782/ (last accessed 5 July 2021).

[r100] Petersen D (2008) Space, time, weight, and flow: Suggestions for enhancing assessment of creative movement. Physical Education & Sport Pedagogy 13(2), pp. 191–198.

[r101] Pfordresher PQ, Halpern AR and Greenspon EB (2015) A mechanism for sensorimotor translation in singing. Music Perception: An Interdisciplinary Journal 32(3), 242–253.

[r138] Piana S, Maneini M, Camurri A, Varni G and Volpe G (2013) Automated Analysis of Non-Verbal Expressive Gesture. Atlantis Ambient and Pervasive Intelligence, 41–54. 10.2991/978-94-6239-018-8_3

[r102] Powers HS and Widdess R (2001) India, Subcontinent of. The New Grove Dicitionary of Music and Musicians. London: Macmillan.

[r103] Rahaim MJ (2009) Gesture, Melody, and the Paramparic Body in Hindustani Vocal Music. Berkeley: University of California.

[r104] Rasamimanana NH, Flety E and Bevilacqua F (2006) Gesture analysis of violin Bow strokes. Lecture Notes in Computer Science 3881, 145–155.

[r105] Reybrouck M (2012) Musical sense-making and the concept of affordance: An ecosemiotic and experiential approach. Biosemiotics 5(3), 391–409.

[r106] Richter M and Wright RA (2014) Contemporary perspectives on effort: A special issue. Motivation and Emotion 38, 745–747.

[r107] Rosenbaum DA (2009) Human Motor Control. San Diego: Academic Press.

[r108] Ryan J (1991) Some remarks on musical instrument design at STEIM. Contemporary Music Review 6(1), 3–17.

[r134] Samadani AA, Burton S, Gorbet R and Kulie D (2013) Laban Effort and Shape Analysis of Affective Hand and Arm Movements. 2013 Humaine Association Conference on Affective Computing and Intelligent Interaction. 10.1109/acii.2013.63

[r109] Schiavio A (2014) *Music in (en) Action. Sense-Making and Neurophenomenology of Musical Experience.* Doctoral dissertation, University of Sheffield.

[r110] Schubert E (2001) Correlation analysis of continuous emotional response to music: Correcting for the effects of serial correlation. Musicae Scientiae 5(1_suppl), 213–236.

[r111] Souza L and Freire S (2017) Gestures of body joints, musical pulses and Laban effort actions: Towards an interactive tool for music and dance. In Proceedings of the 16th Brazilian Symposium on Computer Music, São Paulo, Brazil, pp. 49–54.

[r112] Steele J (2020) What is (perception of) effort? Objective and subjective effort during task performance. PsyArXiv.

[r113] Stergiou N and Decker LM (2011) Human movement variability, nonlinear dynamics, and pathology: Is there a connection? Human Movement Science 30(5), 869–888. 21802756 10.1016/j.humov.2011.06.002 PMC3183280

[r114] Stern DN (2010) Forms of Vitality: Exploring Dynamic Experience in Psychology, the Arts, Psychotherapy, and Development. New York: Oxford University Press.

[r115] Tanaka A (2015) Intention, effort, and restraint: The EMG in musical performance. Leonardo 48(3), 298–299.

[r116] Tanaka A, Altavilla A and Spowage N (2012) Gestural musical affordances. In Proceedings of the 9th Sound and Music Computing Conference, Copenhagen, Denmark, pp. 318–325.

[r117] Tanaka A and Ortiz M (2017) Gestural musical performance with physiological sensors, focusing on the electromyogram. In Lesaffre M, Maes P-J and Leman M (eds), The Routledge companion to embodied music interaction. New York: Routledge, pp. 420–428. 10.4324/9781315621364-2

[r118] van der Meer W and Rao S (2006) What you hear isn’t what you see: The representation and cognition of fast movements in Hindustani music. In Proceedings of the International Symposium Frontiers of Research on Speech and Music, pp. 12–20.

[r119] Varela F, Thompson E and Rosch E (1991) The Embodied Mind: Cognitive Science and Human Experience. Cambridge, MA: MIT Press.

[r120] Vertegaal R, Kieslinger M and Ungvary T (1996) Towards a musician’s cockpit: Transducers, feedback and musical function. Quarterly Progress and Status Report/Speech, Music and Hearing 37, 29–32.

[r121] Volpe G (2003) *Computational Models of Expressive Gesture in Multimedia Systems.* Doctoral thesis, University of Genova, Genova, Italy. Retrieved from http://theses.eurasip.org/media/theses/documents/volpe-gualtiero-computational-models-of-expressive-gesture-in-multimedia-systems.pdf (last accessed 5 July 2021).

[r122] Wanderley MM and Battier M (2000) Trends in Gestural Control of Music (CDROM). Paris: Ircam.

[r123] Ward N (2013) *Effortful Interaction: A New Paradigm for the Design of Digital Musical Instruments.* Doctoral thesis, Queens University Belfast, Belfast, Northern Ireland, UK. Retrieved from http://ethos.bl.uk/OrderDetails.do?uin=uk.bl.ethos.602967 (last accessed 5 July 2021).

[r124] Ward N, Ortiz M, Bernardo F and Tanaka A (2016) Designing and measuring gesture using Laban movement analysis and electromyogram. In Proceedings of the 2016 ACM International Joint Conference on Pervasive and Ubiquitous Computing: Adjunct. New York: Association for Computing Machinery, pp. 995–1000.

[r125] Warren WHJ and Verbrugge RR (1984) Auditory perception of breaking and bouncing events: A case study in ecological acoustics. Journal of Experimental Psychology. Human Perception and Performance 10(5), 704–712. 6238128 10.1037//0096-1523.10.5.704

[r126] Westbrook A and Braver TS (2015) Cognitive effort: A neuroeconomic approach. Cognitive, Affective, & Behavioral Neuroscience 15(2), 395–415. 10.3758/s13415-015-0334-y PMC4445645 25673005

[r127] Widdess R and Sanyal W (2004) Dhrupad: Tradition and Performance in Indian Music (SOAS Musicology Series). Aldershot: Ashgate.

[r128] Winkler T (1995) Making motion musical: Gesture mapping strategies for interactive computer music. In Proceedings of the 1995 International Computer Music Conference, Banff, Alberta, Canada, pp. 261–264.

[r129] Wisneski C and Hammond E (1998) Multi-parameter controllers for audio mixing. In CHI 98 Conference Summary on Human Factors in Computing Systems, pp. 299–300.

[r130] Yu R and Bowman DA (2018) Force push: Exploring expressive gesture-to-force mappings for remote object manipulation in virtual reality. Frontiers in ICT 5, 25.

[r131] Zbikowski LM (2002) Conceptualizing Music: Cognitive Structure, Theory, and Analysis. Oxford: Oxford University Press.

[r132] Zhao L (2001) *Synthesis and Acquisition of Laban Movement Analysis Qualitative Parameters for Communicative Gestures.* Doctoral thesis, University of Pennsylvania, Philadelphia, USA. Retrieved from https://pdfs.semanticscholar.org/f146/2519c9473867afa914674ce49796b7e4d3e5.pdf (last accessed 5 July 2021).

[r152] Zhao L and Badler NI (2005) Acquiring and validating motion qualities from live limb gestures. Graphical Models 67(1), 1–16. 10.1016/j.gmod.2004.08.002

